# Metagenomic signatures reveal the key role of phloretin in amelioration of gut dysbiosis attributed to metabolic dysfunction-associated fatty liver disease by time-dependent modulation of gut microbiome

**DOI:** 10.3389/fmicb.2023.1210517

**Published:** 2023-09-07

**Authors:** Jyoti Chhimwal, Prince Anand, Priyanka Mehta, Mohit Kumar Swarnkar, Vikram Patial, Rajesh Pandey, Yogendra Padwad

**Affiliations:** ^1^Pharmacology and Toxicology Laboratory, Dietetics and Nutrition Technology Division, CSIR-Institute of Himalayan Bioresource Technology (CSIR-IHBT), Palampur, India; ^2^Academy of Scientific and Innovative Research (AcSIR), Ghaziabad, India; ^3^INtegrative GENomics of HOst-PathogEn (INGEN-HOPE) Laboratory, CSIR-Institute of Genomics and Integrative Biology (CSIR-IGIB), New Delhi, India; ^4^Biotechnology Division, CSIR-Institute of Himalayan Bioresource Technology (CSIR-IHBT), Palampur, India

**Keywords:** gut microbiome, MAFLD, phloretin, 16S rRNA, metagenome

## Abstract

The importance of gut-liver axis in the pathophysiology of metabolic dysfunction-associated fatty liver disease (MAFLD) is being investigated more closely in recent times. However, the inevitable changes in gut microbiota during progression of the disease merits closer look. The present work intends to assess the time-dependent gut dysbiosis in MAFLD, its implications in disease progression and role of plant-derived prebiotics in its attenuation. Male C57BL/6J mice were given western diet (WD) for up to 16 weeks and phloretin was administered orally. The fecal samples of mice were collected every fourth week for 16 weeks. The animals were sacrificed at the end of the study and biochemical and histological analyses were performed. Further, 16S rRNA amplicon sequencing analysis was performed to investigate longitudinal modification of gut microbiome at different time points. Findings of our study corroborate that phloretin alleviated the metabolic changes and mitigated circulating inflammatory cytokines levels. Phloretin treatment resists WD induced changes in microbial diversity of mice and decreased endotoxin content. Prolonged exposure of WD changed dynamics of gut microbiota abundance and distribution. Increased abundance of pathogenic taxa like *Desulfovibrionaceae, Peptostreptococcus, Clostridium*, and *Terrisporobacter* was noted. Phloretin treatment not only reversed this dysbiosis but also modulated taxonomic signatures of beneficial microbes like *Ruminococcus*, *Lactobacillus*, and *Alloprevotella*. Therefore, the potential of phloretin to restore gut eubiosis could be utilized as an intervention strategy for the prevention of MAFLD and related metabolic disorders.

## 1. Introduction

The global burden of metabolic dysfunction-associated fatty liver disease (MAFLD) or previously called non-alcoholic fatty liver disease (NAFLD) is a pharmacological challenge with a prevalence of 21–30% among adults worldwide (Mitra et al., 2020). NAFLD could eventually lead to non-alcoholic steatohepatitis (NASH), associated with fibrosis, cirrhosis, and finally to hepatocellular carcinoma (HCC), which is a foremost cause of liver-related morbidity and mortality (Chhimwal et al., 2021). Both, NAFLD and NASH are emerging as a modern public health concern. The association of NAFLD or MAFLD with other life style disorders viz. obesity, type 2 diabetes and cardiovascular diseases contribute toward morbidity and mortality related to liver disease (Ismaiel and Dumitraşcu, 2019). The opportunities for the right treatment regimen are still evolving and development of safe and targeted pharmacotherapy is possibly the utmost need for the treatment of MAFLD. The molecular basis of MAFLD development is not well understood but the “multiple hit” concept emphasizes the role of gut microbiota, insulin resistance, and adipokines produced by adipose tissues which generally attributed to the occurrence of dysfunctional lipid metabolism, oxidative stress and hepatic inflammation (Ji et al., 2019).

The number of bacteria in the stomach is estimated to be 10 times that of human cells (over 10^14^) (Ji et al., 2019). The complex relationship of gut microbes and human host is reciprocally beneficial but at the same time, gut dysbiosis is linked with different chronic metabolic diseases, including MAFLD (Fan and Pedersen, 2021). Gut microbiome has attracted considerable attention due to its crucial role in the pathogenesis and development of MAFLD via the gut-liver axis. The liver health is highly reliant on the gut health, as any disruptions in the intestinal barrier expose liver to the pathogenic microorganisms and their toxic byproducts (De Muynck et al., 2021). Disturbance in gut microbiota leads to increased abundance of harmful microbes like endotoxin and ethanol producing bacteria and production of substances like choline and trimethylamine from its precursor phosphatidylcholine (Lee et al., 2020a). The patients with MAFLD also been reported having altered microbial populations in the gut (Tsai et al., 2020). Nevertheless, the impact of liver and gut microbiome and its dysbiosis on MAFLD biology and its potential interactions with the host phenome are largely unexplored, which confines our mechanistic understanding of metagenomic association with the disease (Sookoian et al., 2020). Additionally, majority of the studies of gut microbiota in MAFLD solely pertain on the endpoints and discuss the culminating changes occurred after the progression of the disease. Efforts toward comprehending the sequential changes in gut dysbiosis during progression of MAFLD would be useful to elucidate and essentially a focus of this manuscript.

Diet appears to be a crucial determinant in regulating gut microbiota composition, which is implicated in several metabolic processes including MAFLD (Quesada-Vázquez et al., 2020). The gut microbiota plays a role in the extraction of calories from diet and regulates fat storage. An unbalanced microbiome can result in excessive calorie extraction and fat accumulation (Davis, 2016). Previous studies have established a correlation between alterations in the gut microbiota caused by the western diet (WD) and the development of MAFLD (Romualdo et al., 2022; Yang et al., 2023). However, the paucity of long-term dietary studies or interventions with repeated fecal sample timepoints and post-intervention follow-ups limits current knowledge of how dietary habits alter the gut microflora and its impact on MAFLD progression. Therefore, the present study was conducted to better understand the correlation between WD induced gut dysbiosis and MAFLD progression utilizing a time-dependent approach, as well as implications of this finding for possible therapeutic approaches. Many dietary, phytomolecules and prebiotic-based strategies have been reported in promoting healthy gut microbiota, growth and associated benefits (Carrera-Quintanar et al., 2018; Beane et al., 2021). Phloretin, a dihydrochalcone flavonoid derived from apples or pears, is one such nutraceutical that has been shown to alter the gut microbiota associated with colitis (Wu et al., 2019). Phloretin is also effective in ameliorating MAFLD by regulating AMP-activated protein kinase (AMPK) pathways (Liou et al., 2020). In our previous study, we discussed how phloretin restored hepatic autophagy to slow the course of MAFLD (Chhimwal et al., 2022). However, it would be interesting to see if the modulation of gut microbiota could be attributed to the efficacy of orally administered phloretin in ameliorating MAFLD. In this study, taking advantage of the susceptibility of gut microbiota to over-nutrition, we looked at how the gut microflora changes over the course of 16 weeks of WD feeding. Further, we explored the potential of phloretin to maintain metabolic homeostasis while altering the gut microflora associated with MAFLD over time.

## 2. Materials and methods

### 2.1. Animals used in the study

Six-weeks-old, adult male C57BL/6J mice, weighing 25 ± 3 g were procured from CSIR-Indian Institute of Integrative Medicine, Jammu, India. The animals were caged into individually ventilated cages (IVC) under standard laboratory temperature and humidity conditions and allowed free access to standard mouse chow pellet feed and water *ad libitum*. Both animal handling procedures and protocols were approved by the Institutional Animal Ethical Committee (IAEC) and carried out as per the guidelines of Committee for the Purpose of Control and Supervision of Experiments on Animals (CPCSEA).

### 2.2. Experimental design

C57BL/6J mice were randomly separated into three groups (*n* = 6).

1. *Group 1 (Control):* The animals of this group were provided standard pellet feed and served as control group.
2. *Group 2 (WD):* The animals were fed on WD (44.4% kcal from carbohydrates, 40.1% kcal from fat, supplemented with 0.15% cholesterol) along-with HFCS (high fructose corn syrup, 42%) in drinking water for 16 weeks.
3. *Group 3 (WD + P):* The animals received phloretin [dissolved in 0.5% sodium carboxymethyl cellulose (CMC-Na) solution] by oral gavage at 200 mg/kg body weight dose along with WD and HFSC water. The dose of phloretin was optimized using previous research (Chhimwal et al., 2022).

**FIGURE 1:**
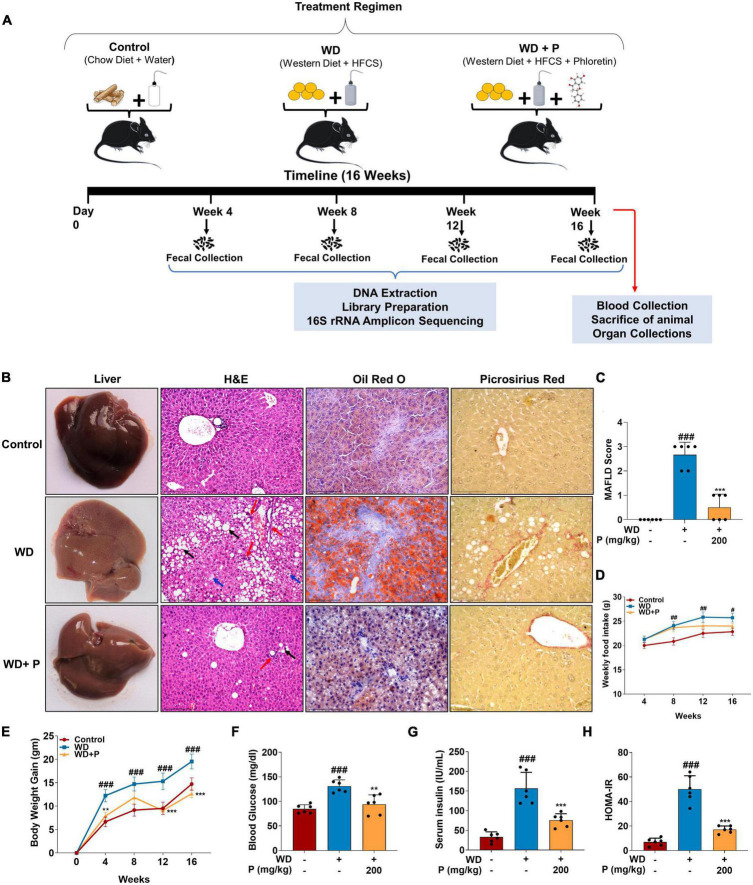
Phloretin improved pathological injury, hepatic lipid deposition and insulin resistance in western diet (WD) fed mice (*n* = 6). **(A)** Schematic diagram illustrating treatment regimen. **(B)** Representative images of liver along with H&E (scale bars, 200 μm; black arrows represent macrosteatosis, blue arrows represent microsteatosis, and red arrows represent inflammatory focci), Oil Red O staining (scale bars, 100 μm) from each group and Picrosirius red staining (scale bars, 100 μm; red color represent fibrous tissues). **(C)** Graphical representation of semi-quantitative scoring of MAFLD in different groups. **(D)** Graphical representation of weekly food intake in different groups. **(E)** Graphical representation of body weight gain in different groups. **(F)** Blood glucose level in different groups. **(G)** Serum insulin level in different groups. **(H)** Graphical representation of HOMA-IR in all groups. The symbols “#,” “##,” “###” represent *p* < 0.05, *p* < 0.01, and *p* < 0.001, respectively vs. control group; the symbols “**,” “***” represent *p* < 0.01, *p* < 0.001, respectively vs. WD group.

Figure 1A illustrates the overall treatment regimen. Mice in control and WD group receive equal volume of CMC-Na solution orally to normalize the handling stress. The fecal samples of mice were collected every fourth week. The body weight and food intake of mice were measured weekly. At the end of the study, the animals were euthanized by CO_2_ asphyxiation and the liver tissues were excised after the dissection of animals and stored for further analysis.

### 2.3. Serum biochemistry

Serum triglyceride (TG), total cholesterol (TC), low-density lipoprotein cholesterol (LDL-C), and high-density lipoprotein cholesterol (HDL-C) levels were determined by colorimetry based automated bioanalyzer using designated colorimetric kits (ERBA, Transasia, India), as per manufacturer’s protocol.

### 2.4. ELISA for serum insulin and inflammatory cytokines

The serum concentrations of insulin and inflammatory cytokines (IL-6 and TNF-α) were assessed using designated ELISA kits (RayBiotech, GA, USA), as per manufacturer’s instructions.

### 2.5. Estimation of fecal endotoxin level

To determine the fecal bacterial endotoxin concentration, 200 mg of fecal samples were homogenized and further sonicated in 1 ml of PBS. The supernatants were collected and subjected to endotoxin estimation using commercially available chromogenic endotoxin ELISA kit (MyBioSource, CA, USA) following manufacturer’s instructions.

### 2.6. Histopathology and ORO staining in liver tissue

Formalin-fixed liver tissues were processed for histopathology as described by Chhimwal et al. (2020) and then sliced into 4 μM thick section to perform hematoxylin and eosin (H&E) and Picrosirius red staining following procedure reported previously (Chhimwal et al., 2020). Further, snap frozen liver specimens were embedded in OCT medium and sliced at 10 μm thickness with the help of cryotome (Lieca, Wetzlar, Germany). Followed by fixation in 10% buffered formalin for 10 min, and rinsed in water for 5 min. Fixed sections were then stained with freshly prepared 0.4% ORO staining solution for 10 min at RT followed by counter stain with Mayer’s hematoxylin and again washed with running water for 5 min. Stained liver sections were then envisioned under bright field in EVOS FL Auto 2 microscope (Thermo Fisher Scientific, MA, USA). MAFLD scoring was done according to previously described method (Chhimwal et al., 2021).

### 2.7. RNA isolation and RT-qPCR

Total RNA was isolated from liver tissue using TRIzol TRI Reagent (Sigma, MO, USA). Briefly, 50 mg of liver tissue was homogenized in one ml of TRI reagent and the supernatant was collected by centrifugation at 12,000 *g* for 5 min at 4°C. To separate the aqueous layer containing nucleic acids, 0.2 ml chloroform was added per ml of collected supernatant and centrifuged (12,000 *g*; 15 min; 4°C). The upper aqueous layer was collected and an equal volume of 70% ethanol was added to it. The mixture was then passed through the column provided in RNeasy Mini Kit (Qiagen, Hilden, Germany) and further RNA was isolated using the manufacturer’s protocol. The quality and quantity of RNA samples were checked by NanoDrop (Thermo Fisher Scientific, MA, USA). RT-qPCR reactions were executed using RT-qPCR ROX Mix Kit supplied by Thermo Fisher Scientific. GAPDH was used as reference gene. Primers sequences (forward and reverse) are listed in Supplementary Table 1. All the data (analyzed by 2^–ΔΔ*Ct*^ method) were adjusted to reference gene and displayed as fold change versus the control group.

### 2.8. DNA isolation, library preparation, and 16S rRNA sequencing

Total bacterial DNA was isolated from all 72 samples using QIAamp PowerFecal Pro DNA Kit (Qiagen, Hilden, Germany) according to manufacturer’s instructions. The quality and quantity of DNA samples were checked by Qubit 2.0 fluorometer (Thermo Fisher Scientific, MA, USA). The amplicon PCR was carried out using the primers 341F (5′-CCTACGGGNGGCWGCAG-3′) and 805R (5′-GACTACHVGGGTATCTAATCC-3′) to amplify the V3–V4 region of the bacterial 16S rRNA gene. Following the PCR purification, the Nextera XT indexed adapters (Illumina, CA, USA) were added to the ends of the primers. KAPA HiFi HotStart ReadyMix (2X) (Roche, Basel, Switzerland) was used for all PCR reactions. The purified libraries were then subjected to quality check on Qubit 2.0 fluorometer (Thermo Fisher Scientific, MA, USA) and Agilent 2100 Bioanalyzer system (Agilent, Singapore). The libraries were then sequenced and 250 bp paired-end reads were generated on the Illumina platform using MiSeq Reagent Kit v3 (600 cycle) (Illumina, CA, USA). By sequencing all the samples in a single MiSeq run, any potential batch effects were eliminated.

### 2.9. Processing of raw sequencing data

Computing and data processing was performed using the computing resource on Google cloud platform. Trimming of raw reads was done by remove primers using cutadapt (Martin, 2011) and low-quality bases (<Q30). These high-quality reads were considered for denoising, merging of paired-end reads, removal of chimeric sequences, and to pick unique amplicon sequence variants (ASVs) using the DADA2 plugin within qiime2 (Caporaso et al., 2010; Callahan et al., 2016). For taxonomic assignments of these ASVs, Silva database (sequences along with taxonomy, version silva-138-99) were downloaded (Quast et al., 2013). Further, unclassified ASVs from the same family, genus or species were differentiated using consecutive numbering (1, 2, 3, and so on). Selected sequences, extracted using V3–V4 primers, were used to train the naïve Bayes classifier and this trained classifier was used to predict the complete taxonomy of each ASV. This training and testing were done using feature-classifier fit-classifier-naive-bayes, and feature-classifier classify-sklearn plugins within qiime2. Furthermore, functional potential of these microbial communities was evaluated using Phylogenetic Investigation of Communities by Reconstruction of Unobserved States (PICRUSt, version 2; maximum nearest sequenced taxon index = 2) (Langille et al., 2013). Abundance of each ASV and predicted KEGG (kyoto encyclopedia of genes and genomes) metabolic pathways were used for downstream statistical analysis (Kanehisa and Goto, 2000).

### 2.10. Statistical analysis

One-way or two-way (wherever applicable) ANOVA was performed to analyze data using GraphPad Prism statistical analysis software (version 8.0.2), in order to determine the statistical significance of the differences among groups. Data are represented as mean ± SD (*n* = 6) and *p*-value < 0.05 was considered as significant. R statistical interface was used to perform all microbial community ecology analyses. Because of low sequencing depth in 2 out of total of 72 samples, 70 samples were considered for downstream analysis. ASV counts were used to access the microbial richness, diversity and ASVs relative abundances were used to calculate distances between each sample using vegan and ape packages in R (Paradis and Schliep, 2019). Bray–Curtis distances were used for the ordination analysis. Adonis function in R was used to calculate permutational multivariate analysis of variance (PERMANOVA) for each variable. To identify significantly discriminating taxa and pathways, “indicator species analysis” was performed using labdsv package and variance partition analysis performed using Variance Partition package in R (Hoffman and Schadt, 2016; Roberts et al., 2019). Indicator species analysis (ISA) is one of the most used statistical methods in microbial ecology. The benefit of using ISA is that it provides the features (taxa/pathways) which are significantly discriminating across groups (*p*-value) and at the same time their prevalence within the group (IndVal score). Cumulative abundances of selected taxa and functions were shown in different plots. Kruskal–Wallis test was applied to check the statistical significance of discriminating features (taxa/pathways). To understand the co-occurrence of microbial taxa among all three experimental groups, compositional corrected correlations were performed using Compositionality Corrected by REnormalization and PErmutation (CCREPE) function in R (Schwager et al., 2019). All strong (*r* > 0.5) significant correlations (*p* < 0.05) were used to construct the co-occurrence network plots in Cytoscape (Shannon et al., 2003). Neighborhood Connectivity was used to access the overall network topology.

## 3. Results

### 3.1. Western diet contributed to MAFLD onset by triggering lipid accumulation, pathological, and fibrotic changes in liver and altering metabolic homeostasis

Effects of WD on pathophysiology of animal and induction of MAFLD has been evaluated through different parameters. ORO staining in Figure 1B shows that WD-fed mice had significant micro and macro-vesicular fat deposition in the hepatocytes. The H&E staining of liver sections revealed ballooning degeneration along with multifocal necrotic regions and invasive mononuclear cells indicating hepatic inflammation (*p* < 0.001; Figure 1C). In comparison to the control group, WD feeding resulted in mild to moderate fibrous tissue deposition surrounding steatotic cells and vessels. We also observed a significant increase in feed intake in WD-fed mice over the course of 16 weeks. This resulted in an average 20 g of weight gain in WD mice, which is more than 30% of the weight of control mice (*p* < 0.001) (Figures 1D, E). Further, we observed that the blood glucose and serum insulin levels were significantly elevated (*p* < 0.001) in WD fed mice (Figures 1F, G).

### 3.2. Phloretin reduced pathological injury, hepatic fat deposition, fibrosis, blood glucose, and insulin resistance in WD fed mice

Treatment with phloretin substantially reduced the fat deposition in the liver while preserving normal hepatic architecture (Figure 1B). In comparison to the WD group, phloretin administration was observed to significantly (*p* < 0.001) decrease the extent of degenerative alterations in the liver (Figures 1B, C). Administration of phloretin also lowered the fibrotic changes in the liver as compared to the WD group. Furthermore, as compared to the WD group, we observed a 40% reduction (*p* < 0.001) in body weight gain of phloretin treated mice, however, no significant change was observed in weekly food intake (Figures 1D, E). Phloretin treatment also resulted in a significant (*p* < 0.01, *p* < 0.001, respectively) reduction in blood glucose and serum insulin levels thereby maintaining homeostasis model assessment-estimated insulin resistance (HOMA-IR) (Figures 1F–H).

### 3.3. Continuous exposure to WD resulted in dyslipidemia, overexpression of lipogenic marker genes, inflammation, and increased fecal endotoxin level as disease progresses

**FIGURE 2:**
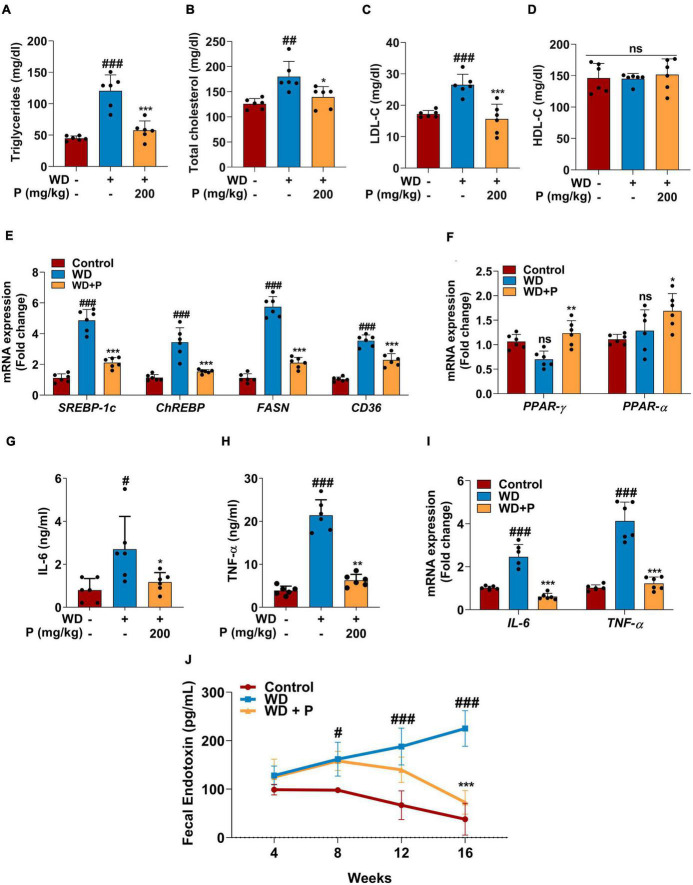
Phloretin maintained serum lipid profile, metabolic homeostasis and decreased the inflammatory markers in WD fed mice (*n* = 6). Panels **(A–D)** denote the impact of phloretin on serum triglycerides, cholesterol, LDL-C, and HDL-C level, respectively. **(E)** Quantitative representation of the gene expression of *SREBP-1c*, *ChREBP*, *FASN*, and *CD36*, respectively in each group. **(F)** Quantitative representation of the gene expression of *PPAR*-γ and *PPAR*-α, respectively in each group. Panels **(G,H)** represent the serum concentrations of IL-6 and TNF-α level, respectively. **(I)** mRNA expression of *IL-6* and *TNF*-α in liver tissue from different groups. **(J)** Fecal endotoxin level in all group at different time points. The symbols “#,” “##,” “###” represent *p* < 0.05, *p* < 0.01, *p* < 0.001, respectively vs. control group; the symbols “*,” “**,” “***” represent *p* < 0.05, *p* < 0.01, *p* < 0.001, respectively vs. WD group; ns represents non-significant.

Western diet feeding resulted in dyslipidemia causing significant increase in serum triglycerides (TG) and cholesterol (TC) levels. Precisely, following 16 weeks of WD feeding, a ∼3-fold (*p* < 0.001) increase in TG and ∼1.5-fold increase in both TC (*p* < 0.01) and LDL-C (*p* < 0.001) levels were observed (Figures 2A–C). Abnormal lipid metabolism is a defining feature of MAFLD, which is accompanied by alterations in the lipid metabolism pathway. Thus, we investigated the expression of genes encoding for fatty acid (FA) metabolism to govern the effect of phloretin on hepatic lipid metabolism. Our findings showed that the hepatic expression levels of lipogenic genes *SREBP-1c*, *ChREBP*, *CD36*, and *FASN* are elevated in response to WD, which contributes to *de novo* lipid synthesis (Figure 2E). In comparison to the control group, a 4.8-fold (*p* < 0.001) and a 3.4-fold (*p* < 0.001) elevation in the mRNA expression of *SREBP-1c* and *ChREBP* transcripts, respectively, as well as a 5.7-fold (*p* < 0.001) increase in the FASN gene expression was detected in the liver of WD group mice. Apart from an increase in *SREBP-1c*, *ChREBP*, and *FASN*, a 3.5-fold (*p* < 0.001) rise in the mRNA expression of *CD36* was also observed in WD fed mice. Further, in WD fed mice, the mRNA expression of *PPAR*-γ and *PPAR*-α showed a small but no significant alteration (Figure 2F).

Steatosis-induced inflammation plays a critical role in the progression of disease to more severe outcomes. Serum ELISA has been performed to evaluate the levels of inflammatory cytokine markers. Results revealed that exposure to WD augmented the circulating inflammatory cytokines viz. IL-6 (>3-fold; *p* < 0.05) and TNF-α (∼5-fold; *p* < 0.001) levels (Figures 2G, H). Further, the gene expression analysis revealed significant (*p* < 0.001; Figure 2I) elevation in *IL-6* and *TNF*-α expression after chronic exposure to WD. Additionally, Fecal endotoxin levels play critical role in assisting the inflammation and disease progression. The fecal endotoxin level was estimated in each group every fourth week. At week 4, a slightly elevated but not significant increase was observed in endotoxin level in feces of mice from WD and WD + P groups. However, continuous exposure to WD led to a substantial increase in the endotoxin release at 8th, 12th, and 16th week (*p* < 0.05, *p* < 0.001, *p* < 0.001, respectively) as compared to the control group (Figure 2J).

### 3.4. Phloretin improved abnormal lipid metabolism by modulating the expression of metabolism related genes

Phloretin treatment was found to improve lipid homeostasis and help in subverting the dyslipidemia in WD-fed mice. As illustrated in Figure 2, phloretin treatment of WD-fed mice prevented increase in TG (*p* < 0.001), TC (*p* < 0.01), and LDL-C (*p* < 0.001) levels (Figures 2A–C). However, no statistically significant difference was observed in the levels of HDL-C across all the groups (Figure 2D). Moreover, phloretin treatment reduced the expression of lipogenic marker genes. Importantly, we observed a 57 and 56% reduction (*p* < 0.001) in the expression of *SREBP-1c* and *ChREBP*, respectively on treatment with phloretin. Similarly, phloretin treatment resulted in a 2.7-fold (*p* < 0.001) and ∼1.6-fold (*p* < 0.001) downregulation of *FASN* and *CD36* genes, respectively (Figure 2E). Interestingly, phloretin supplement further upregulated the mRNA expression of *PPAR*-γ and *PPAR*-α by 1.7-fold (*p* < 0.01) and 1.3-fold (*p* < 0.05) respectively, facilitating hepatic FAs oxidation (Figure 2F).

### 3.5. Phloretin improved hepatic inflammation by mitigating inflammatory cytokines (IL-6 and TNF-α) levels and subverting the fecal endotoxin levels

On the contrary to the WD exposure, phloretin treatment significantly reduced the levels of inflammatory cytokine markets IL-6 and TNF-α their normal levels (Figures 2G, H). Phloretin, in instance, was found to cause a 2.3-fold (*p* < 0.05) and >3-fold (*p* < 0.01) decline in serum IL-6 and TNF-α levels, respectively. The gene expression of *IL-6* and *TNF*-α further demonstrated the ability of phloretin to diminish hepatic inflammation and consequently prevent the progression of NASH in mice (*p* < 0.001; Figure 2I). Furthermore, the endotoxin release initially increased in the feces of mice treated with phloretin, but prolonged exposure to phloretin decreased the endotoxin level to normal (Figure 2J). The result indicated the protective efficacy of phloretin against microbial toxic byproducts. These results suggested the potential of phloretin in mitigation of WD-induced imparities leading to MAFLD progression in mice.

### 3.6. Gut microbial diversity changes induced by prolonged exposure of WD and phloretin supplementation

**FIGURE 3:**
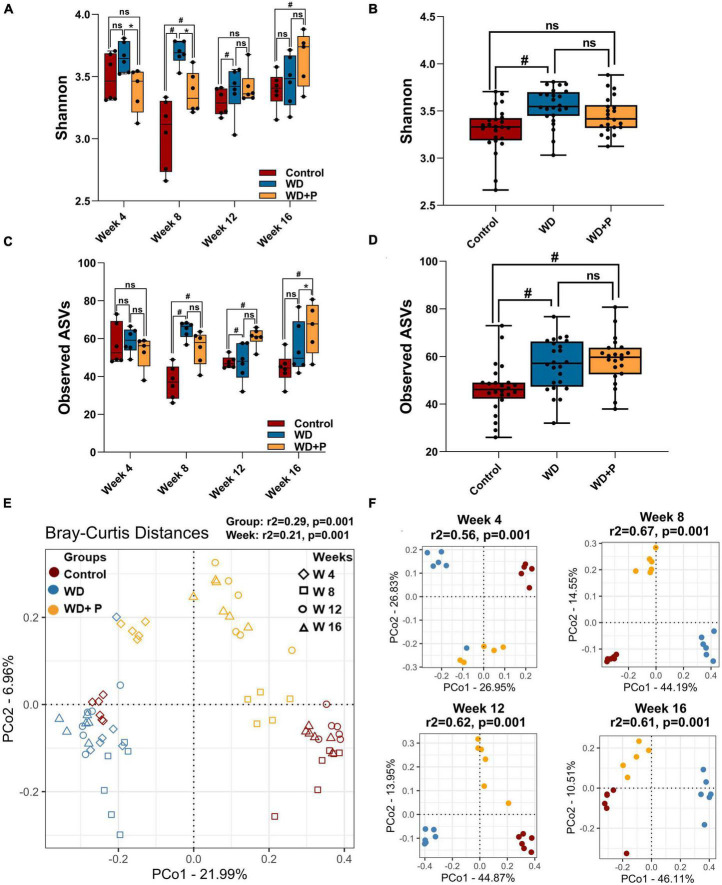
Microbial alpha and beta diversity was observed across different experimental groups (*n* = 6). **(A)** Microbial diversity among different treatment groups across four time points, **(B)** overall diversity across different treatment independent of time **(C)** taxa richness among different treatment groups across four time points, **(D)** overall richness across different treatment independent of time. The symbol “#” represents *p* < 0.05 vs. control group and the symbol “*” represents *p* < 0.05 vs. western diet (WD) group according to Kruskal–Wallis test; ns represents non-significant. All the alpha diversity measures along with the metadata information are provided in the Supplementary Table 2. **(E,F)** PCoA analysis was performed on Bray–Curtis distances calculated on relative abundance of each species. **(E)** All experimental groups at different time points were considered. This shows the strong differences due to diet (PERMANOVA, *r*^2^ = 0.29) as compared to the timepoints (PERMANOVA, *r*^2^ = 0.21) and **(F)** differences between experimental groups at each time point such as week 4 (top left), week 8 (top right), week 12 (bottom left), and week 16 (bottom right) were observed. Strong differences (PERMANOVA, *r*^2^ = 0.56–0.67) were observed among the treatment groups at each time point.

To evaluate the role of gut-microbes in WD-induced disease progression and role of phloretin as counter strategies 16S rRNA sequencing and analysis of metagenome was performed. Post 16S rRNA sequencing, data was analyzed and microbial diversity was compared across the treatment groups at different time-points (Figure 3). Analysis of comparative microbial diversity and richness within groups at different time intervals revealed differences in diversity, as showed by the Shannon index and observed ASVs (*p* < 0.05). Significant differences were observed in diversity among WD groups versus control group, stratified across time points, prominent change was observed after 8 weeks (Figure 3A). Phloretin treatment reversed these changes at week 4, 8, and 12 but not at week 16. However, phloretin treatment maintained the overall diversity index similar to the control group (Figure 3B). Furthermore, there was a statistically significant difference (*p* < 0.05) in taxonomic richness between control and WD fed groups at week 8, 12, and 16. However, significant change in the richness (*p* < 0.05) between the WD and WD + P groups was observed only at week 12 (Figure 3C). Phloretin did not effectively alter the microbial richness at each time point; however, it managed to reverse the overall richness (Figure 3D). Overall, these results indicated that phloretin treatment resists WD induced changes in microbial diversity and richness in mice and indicates its prophylactic effects to maintain the diversity similar to healthy control group. Detailed Shannon and Simpson indices and observed ASVs of individual samples were given in Supplementary Table 2.

### 3.7. Long-term phloretin administration restored the microbial community structure that had been disrupted by WD feeding

Distance matrices suggest that WD fed mice exhibited distant diversity as compared to control and phloretin treatment group, which was comparatively less distant to the control group (Figure 3E). Additionally, separate comparative PCoA analysis at each time point exhibited a prominent difference in microbial diversity of WD as compared to control and WD + P (PERMANOVA *r*^2^ ranging between 0.57 and 0.65) (Figure 3F). Results indicate that Bray–Curtis distance was higher between WD and control group across the timeline. On the other hand, the diversity of the phloretin treatment group was comparatively less distant to control group. These outcomes substantiated that phloretin treatment restores the changes in microbial community composition.

### 3.8. Chronic WD exposure and phloretin supplementation both affected the dynamics of gut microbiota abundance and distribution

**FIGURE 4:**
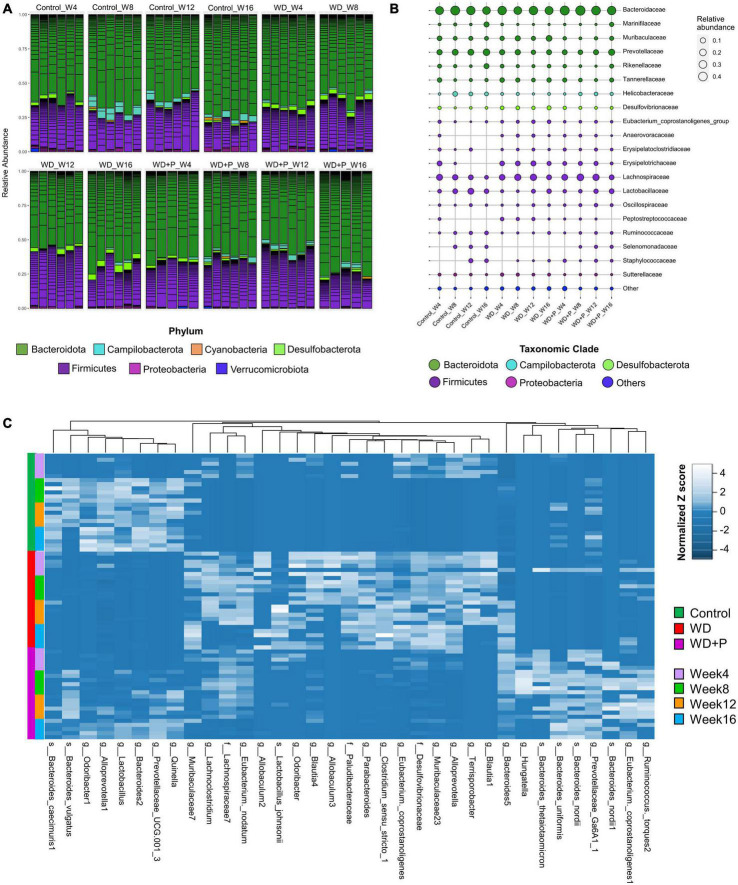
Taxonomic signatures of selected taxa identified in each experimental group (*n* = 6). **(A)** Relative abundance of different bacterial phylum was observed, **(B)** mean relative abundance of top 20 bacterial families were observed, and **(C)** the abundance of selected ASVs using Variance Partition was shown using heatmap signatures, which demonstrated a disparity in their abundance in different group at each time point.

In order to evaluate the phylogenetic comparison of microbial communities, taxonomic signatures were identified in each experimental group at different timepoints. Relative abundance of different phylum and mean relative abundance of top 20 bacterial families were observed (Figure 4). After fourth week, relative abundance of *Firmicutes* was decreased and *Desulfobacterota* increased in the WD group as compared to control. In phloretin treatment group, no such observation was recorded for *Desulfobacterota*. In the eighth week, increase in *Campilobacterota* and decreased abundance of *Desulfobacterota* was observed in the phloretin treatment group similar to control but not in the WD group. After 12th week, *Campilobacterota* had largely decreased abundance in WD group as compared to the control and phloretin treatment groups. In 16th week, abundance of *Firmicutes* was decreased in the WD and phloretin treated groups as compared to the control. Large increase in abundance of *Desulfobacterota* was observed in the WD group, which was negligible in control and phloretin treated groups comparatively. Cumulatively, with increase in time abundance of *Campilobacterota* was increased and *Desulfobacterota* was decreased in the phloretin treatment group similar to control but dissimilar to the WD fed mice. Additionally, *Cyanobacteria* was found to be more abundant in control and phloretin treated mice as compared to control. Interestingly, abundance of *Proteobacteria* was found to be increased with time in the control group but decreased in the WD and phloretin treatment group (Figure 4A).

To delve deeper, mean relative abundance of top 20 bacterial families were analyzed. Abundance of *Muribaculaceae, Tannerellaceae, Desulfovibrionaceae*, and *Lachnospiraceae*, were increased in WD fed mice in comparison to control and phloretin treatment groups. Similarly, *Anaerovoraceae, Peptostreptococcaceae*, and *Erysipelotrichaceae* were least abundant or absent in the control group as compared to WD and phloretin treatment group. *Ruminococcaceae* was less abundant whereas *Selenomonadaceae* was completely absent in WD fed mice as compared to control group. Interestingly, abundance of *Helicobacteraceae* was more abundant in control as compared to the other groups. Although, it is highly unlikely that WDs with comparable nutrients would lead to decrease in *Helicobacteraceae* abundance observed in our study, but it is still a potential limitation of the present study. Finally, decreased abundance of *Lactobacillaceae* was observed after WD intake, which improves with the phloretin treatment (Figure 4B and Supplementary Figure 1). At the same time, pathogenic anaerobes like *Peptostreptococcaceae* were also found abundant in the WD fed group, which was least abundant or absent in the control group. The phloretin treatment group witnessed less abundance of these bacterial families and increased abundance of non-pathogenic *Lactobacillaceae* family (Figure 4B and Supplementary Figure 1). We also checked for the relative abundance of ASVs that had >1% occurrence and was plotted to observe the strain level difference amongst samples. The *Bacteroides-5* genus was observed to be more prevalent in WD group as compared to the control and phloretin treated groups, with an increase in dominance occurring over time. In comparison, ASVs belonging to the species *Bacteroides vulgatus* remained more dominant in the both control and phloretin treated group. Supplementary Figure 2 illustrates the dynamic of the gut bacterial population among the groups over a period of 16 weeks and demonstrate the diet and treatment induced variations in taxa. Taxonomic signature analysis upholds the role of WD in alteration of microbial diversity dynamics and phloretin was found to restore these changes.

### 3.9. Heatmap signature revealed that phloretin reversed the ASVs-specific changes in gut microbiota occurred during progression of disease

Variance Partition analysis was performed to assess differentially abundant taxa in all experimental groups using diet pattern and time as independent variables. The diet pattern appeared to attribute more fraction of the variance as compared to time (weeks) (Supplementary Table 3). Heatmap signature of selected taxa (with a variable fraction value >0.25 of diet pattern) revealed the changes in abundance of observed ASVs at family, genus and species level across the treatment groups (Figure 4C). Hierarchical clustering heatmap revealed association of the selected taxa with the progression of MAFLD and phloretin treatment. On a larger extent, ASVs belonging to the genus *Blautia, Terrisporobacter, Alloprevotella, Muribaculaceae, Eubacterium, Clostridium, Parabacteroides, Allobaculum, Odoribacter*, and family *Desulfovibrionaceae, Paludibacteraceae*, was highly abundant in WD fed mice indicating a positive association with the progression of MAFLD. These taxa have been shown less abundant in control and WD + P groups, which suggests negative correlation with phloretin treatment. Interestingly, *Lactobacillus johnsonii* species were also abundant in the WD group. In contrast, several ASVs belonging to genus *Lactobacillus, Quinella, Prevotellaceae, Alloprevotella-1, Odoribacter-1, Bacteroides-1*, and species *B. vulgatus* and *Bacteroides caecimuris-1* showed negative association with WD intake and they started restoring to their normal abundance status in phloretin treatment group. Additionally, taxa from genus *Ruminococcus, Eubacterium coppostanoligenes 1, Hungatella, Bacteroides-5* and some species like *Bacteroides nordii*, and *Bacteroides uniformis* was highly abundant in WD + P group and was less abundant in both control and WD groups. Interestingly, *Bacteroides thetaiotaomicron* was also found abundant in WD + P group in the early phase but was least abundant in later phases of phloretin treatment. Findings indicates strong positive association of these taxa with phloretin treatment. These associations strongly suggests that WD intake changes the gut microbiota with progression of MAFLD and phloretin treatment not only reversed these changes but also modulated some other important taxa signatures.

### 3.10. Differential bacterial abundance depicted the protective effect of phloretin against MAFLD associated gut dysbiosis

**FIGURE 5:**
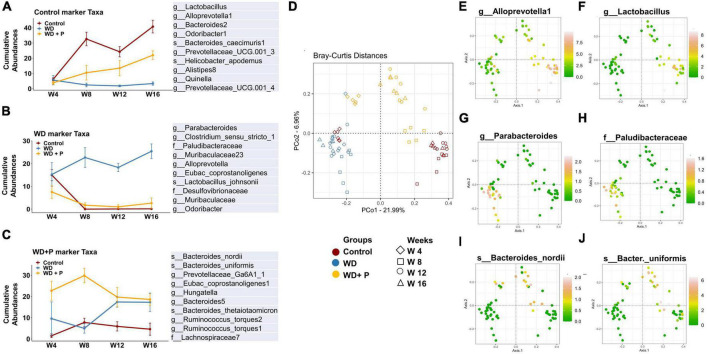
Cumulative relative abundance of taxonomic markers of each experimental group (*n* = 6). Taxonomic signatures were identified using indicator species analysis. Top 10 bacterial species were selected for each experimental group based on the maximum IndVal scores. **(A)** Marker taxa of control group, **(B)** marker taxa of WD group, and **(C)** marker taxa of WD + P. **(D)** Distribution of samples from different experimental group in ordination plot. **(E–J)** Bray–Curtis distances PCoA ordination colored by the relative abundance of selected taxonomic markers of each experimental group. **(E,F)** Distribution of selected taxonomic markers from control group. **(G,F)** Distribution of selected taxonomic markers from WD group. **(I,J)** Distribution of selected taxonomic markers from WD + P group.

To delve deeper into the disease associated changes of microbial taxa signatures, cumulative relative abundance of taxonomic markers and distribution of selected taxonomic markers in ordination plot for each experimental group was evaluated. Taxonomic signatures were identified using ISA. Top 10 bacterial taxa (at families, genera and species level) were selected for each experimental group based on significant discrimination across groups (*p*-value) and their prevalence within the group (the maximum IndVal scores) (Supplementary Table 4 and Figure 5). Clear demarcation between cumulative relative abundance (sum of relative abundances of all 10 representative taxa) was observed across the experimental groups. Results indicate that highly abundant (>35 at week 16) signature marker taxa for control group (belonging to the genus *Lactobacillus, Alloprevotella-1, Bacteroides-2, Prevotellaceae, Odoribacter-1, Alistipes-8*, and *Quinella)* was relatively least abundant in the WD group (<8 at week 16) and was found to be restored after phloretin treatment (>15 at week 16), relative abundance at the start of the study was <10 in all the experimental groups (Figure 5A). Similarly, results suggested relatively high abundance of signature taxa of WD group (belonging to the family *Desulfovibrionaceae, Paludibacteraceae* and genus *Clostridium, Parabacteroides, Muribaculaceae, Odoribacter, Alloprevotella*, and species *L. johnsonii*) was present (>20 at week 16) as compared to the relative abundance of these taxa in control and WD + P group (<5 at week 16) (Figure 5B). Moreover, relative abundance of marker taxa of WD + P group (belonging to family *Lachnospiraceae-7*, genus *Prevotellaceae, Hungatella, Bacteroides*, and species *B. nordii, B. uniformis, Ruminococcus torques 1 and 2)* was decreased with time in WD + P and control groups (>25 at week 8 and <20 at week 16 for WD + P and >5 at week and <5 at week 16 in control) as compared to the WD group, where a time-dependent increment was observed (<10 at week 4 and >15 at week 16) (Figure 5C).

Distribution of selected taxonomic markers in ordination plot was done using PCoA analysis (Figure 5D). Colored ordination plots clearly indicate the higher abundance of ASVs from *Parabacteroides* and *Paludibacteraceae* in the WD group as compared to the control and WD + P groups (Figures 5G, H). Additionally, ASVs from *Lactobacillus, Alloprevotella* were highly abundant in control group and relatively more abundant in the WD + P group as compared to the WD group (Figures 5E, F). *B. nordii* was more abundant in the WD + P group as compared to the WD and control groups and *B. uniformis* was also found to be highly abundant in the WD + P group and week 12 and 16 of control groups as compared to the WD group (Figures 5I, J). It is important to note that all of these observations were largely time dependent, indicating a clear role of phloretin in reversing the effect of gut dysbiosis associated changes in microbial diversity in MAFLD. The samples from each group were then analyzed to create a Spearman’s rank correlation matrix. The selected taxa from each group were used to calculate the distance matrix by Euclidean method. At the end of the fourth week, there was negligible distance between samples indicating all the group sharing similar microbial environment. Furthermore, in the 8th, 12th, and 16th weeks, the control and WD + P groups formed comparable clusters, whereas the WD group formed a distinct cluster (Supplementary Figure 3). The results indicated that the samples in WD group were more distant from the samples in the control and WD + P groups.

### 3.11. Prolonged exposure to WD caused loss of neighborhood connectivity among gut bacterial species

**FIGURE 6:**
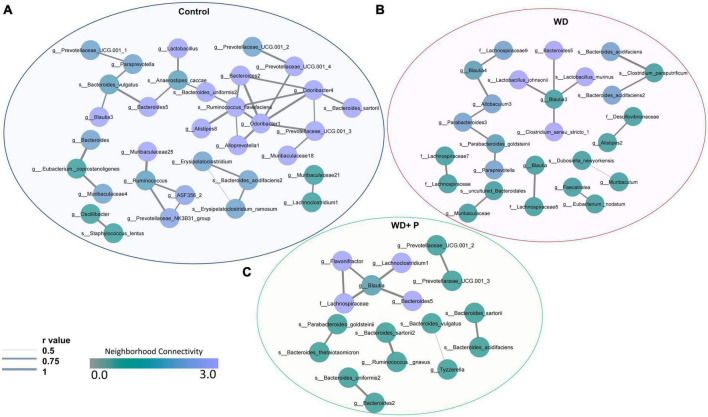
Co-occurrence network analysis using compositionally corrected correlations among different bacterial species (*n* = 6). Network plot generated using the correlation of taxa **(A)** control diet samples, **(B)** WD samples, and **(C)** WD + P diet samples. Nodes represent each bacterial species whereas the thickness of edge represents the correlation strength (*r* value from 0.5 to 1). Color of the node shows how dense the network is based on the neighborhood connectivity.

In order to examine the association between taxa in different groups, a co-occurrence network plot was created with all strong (*r* > 0.5) significant correlations (*p* < 0.05), and only those taxa were picked up that were positively associated to each other in each group. The neighborhood connectivity was found higher in the control group as compared to the WD and WD + P group. In particular, ASVs belonging to genus *Odoribacter-1* was found highly connected with other ASVs from genus *Lactobacillus, Alloprevotella-1, Bacteroides-2, Prevotellaceae, Alistipes-8, Quinella*, and species *Ruminococcus flavefaciens* with *r* value of 1 (Figure 6A). However, absence of *Odoribacter-1* and *R. flavefaciens* in the co-occurrence network of WD and WD + P groups lead to a decrease in neighborhood connectivity. Further, network density of taxa belonging to genus *Blautia-3, Bacteroides-5, Muribaculaceae-25*, and *Muribaculaceae-18* was also higher in the control group with network strength of <1. In contrast, in the WD group the neighborhood connectivity was dense only with taxa of genus *Bacteroides-5, Clostridium_sensu_stricto-1*, and species *Lactobacillus murinus, L. johnsonii* with *r* value of <1 (Figure 6B). However, the neighborhood connectivity was less frequent in the WD + P group as compared to control group but the correlation strength was higher in both control and WD + P group as compared to WD group indicating strong connectivity among bacterial species (Figure 6C). All of these observations indicated loss of neighborhood connectivity as well as network strength among species during the progression of MAFLD. However, phloretin treatment was ineffective in restoring connection, but it did maintain the strength of the network among species.

### 3.12. Functional prediction of gut bacteriome in response to WD and phloretin treatment

**FIGURE 7:**
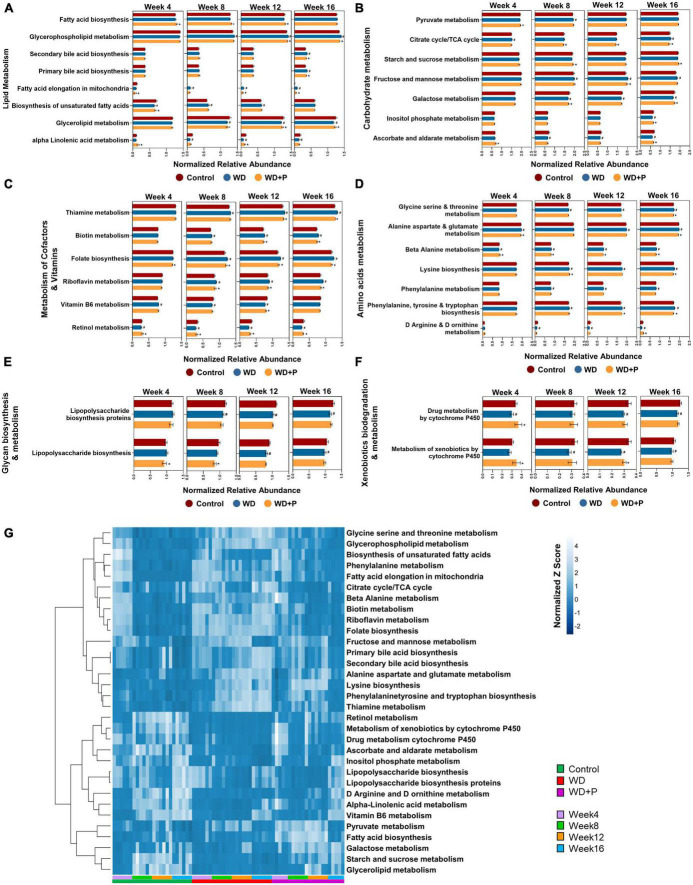
Functional prediction of gut bacteriome in response to WD and phloretin treatment (*n* = 6). Normalized relative abundance of **(A)** lipid metabolism pathways, **(B)** carbohydrate metabolism pathways, **(C)** vitamin metabolism pathways, **(D)** amino acids metabolism pathways, **(E)** glycan biosynthesis and metabolism pathways, **(F)** xenobiotics biodegradation and metabolism pathways in each experimental group. **(G)** Heatmap signature of relative abundance of KEGG metabolism pathway in each group. The symbol “#” represents *p* < 0.05 vs. control group; the symbol “*” represents *p* < 0.05, vs. WD group.

Western diet and phloretin supplementation both have been shown to affect the composition of the gut microbiota, and has potential to cause alterations in bacterial metabolic processes. KEGG pathway signatures were identified in whole microbiome and ISA was performed as a statistical test to determine significantly discriminating metabolic pathways in each group at different time points. A subset of level 3 metabolic pathways that are substantially linked with the course of MAFLD and have a statistically significant (*p* < 0.05) alteration was chosen for each experimental group. WD supplementation resulted in time-dependent enrichment in some of the lipid metabolism pathways, i.e., primary and secondary bile acid biosynthesis, FA elongation in mitochondria, biosynthesis of unsaturated FAs, and glycerophospholipid metabolism. Phloretin supplementation, on the other hand, has been shown to deplete these pathways (Figure 7A). Taking carbohydrate metabolism into account, fructose and mannose metabolism and tricarboxylic acid (TCA) cycle pathways were more abundant in the representative genomes of the taxa possessing a positive association with WD group. In contrast, their abundance appears to be decreased in the phloretin treatment group as compared to WD group. However, galactose metabolism, inositol phosphate metabolism and ascorbate and aldarate metabolism pathways were significantly upregulated by phloretin treatment as compared to WD group (Figure 7B). Vitamin metabolism pathways were also altered in the WD group with time dependent depletion of vitamin B6 and retinol metabolism pathways occurring simultaneously with enrichment of thiamin, biotin, and riboflavin metabolism and the induction of folate biosynthesis. However, it has been demonstrated that phloretin supplementation can help to reverse these alterations (Figure 7C). Amino acids metabolism pathways (excluding D arginine and D ornithine metabolism) were highly enriched in the representative genome from the WD group but less abundant in both control and WD + P groups (Figure 7D). WD group also showed enrichment with pathways involved in lipopolysaccharide biosynthesis at week fourth but depleted with time, as shown by related taxa in glycan biosynthesis and metabolism pathways. However, phloretin supplementation inhibited these pathways as the time progressed further (Figure 7E). Phloretin also facilitates drugs and xenobiotic metabolism by cytochrome P450 pathways in WD fed mice (Figure 7F). Moreover, the heatmap signature demonstrated the abundance of these marker pathways in each sample from each group during time intervals varying from 4th to 16th week (Figure 7G). Since these findings were merely correlations, we can only speculate that altered metabolic pathways were to account for the MAFLD phenotype; nevertheless, more research in this area is required.

## 4. Discussion

Metabolic dysfunction-associated fatty liver disease is becoming increasingly prevalent globally, representing challenge in terms of prevention and treatment. Recent investigation indicated that high fat/calorie rich diet contributed to the development of hepatic steatosis, which progressed to the even more severe NASH following continuous exposure to the diet (Kořínková et al., 2020). Moreover, the consumption of a high-fat/calorie-rich western-style diet causes remodeling of gut microbiota and metabolic dysfunction, which ultimately leads to the advancement of MAFLD or NAFLD (Bakhshimoghaddam and Alizadeh, 2021). However, it would be interesting to understand the dynamic of gut flora as the disease progresses. In light of this fact, the present study demonstrated the longitudinal changes in the gut microbial community as a result of WD feeding along the time course. Moreover, nutraceuticals of natural origin and traditional medicines have recently renewed interest as treatment alternatives in the pharmaceutical industries due to their therapeutic efficacy and fewer side effects. Phloretin is a phenolic dihydrochalcone, found in the apple and has been demonstrated to help prevent diabetes, oxidative injury, and obesity by preserving metabolic homeostasis (Alsanea et al., 2017; Nithiya and Udayakumar, 2017; Shen et al., 2020). Recent report highlighted that intraperitoneally injected phloretin can improve the symptoms of hepatic steatosis caused due to high-fat diet in mice (Liou et al., 2020) although, it would be intriguing to see if its effect on MAFLD is attributed to the remodeling of the gut flora when administered orally.

Findings of the study suggested that mice subjected to WD for an extended period exhibited a broad range of metabolic and histological alterations typical with NASH, including extensive lipid accumulation in the liver, ballooning of hepatocytes, and steatohepatitic foci (Godoy-Matos et al., 2020). However, the treatment of phloretin for 16 weeks was found to be effective in reversing pathological changes in the liver as well as decreasing hepatic lipid accumulation and fibrosis progression (Figure 1B). Insulin resistance is a critical factor in the pathogenesis of NAFLD (Armandi and Schattenberg, 2021). It has been shown previously that prolonged feeding of WD results in rise of fasting blood glucose, serum insulin, increased insulin resistance and other metabolic dysfunctions which ultimately leads to the development of NASH (Choi et al., 2017). Adding support to this, our findings revealed a substantial increase in levels of blood glucose and serum insulin after 16 weeks of WD exposure, indicating the development of insulin resistance. Importantly, phloretin restored the blood glucose and serum insulin levels and maintained the HOMA-IR in WD-fed mice (Figures 1F–H). Increase in circulating glucose and insulin levels associated with insulin resistance have been reported to stimulate *de novo* lipogenesis through the transcriptional regulation of *ChREBP* and *SREBP-1*, respectively (Lee et al., 2021a). The lipogenic marker *SREBP-1c* and its target gene *FASN* found to be upregulated in response to a western style diet, contributing to hepatic *de novo* lipogenesis (Li et al., 2016). Similarly, FA translocase, also known as CD36, is a protein that orchestrates a critical role in enhancing uptake of FAs in hepatocytes as well as some other cell types in the liver, and has been implicated in the advancement of hepatic steatosis (Fu et al., 2021). Phloretin downregulated the mRNA expression of *ChREBP*, *SREBP-1c*, *FAS*, and *CD36* indicating its ability to reduce hepatic *de novo* lipogenesis. Phloretin further contributed to the preservation of metabolic homeostasis by elevating the expression of *PPAR*-α and *PPAR*-γ. Phloretin also maintained the normal lipid profile indicating its protective effect against the dyslipidemia associated with MAFLD (Figures 2A–D). These findings were further corroborated by Liou et al. (2020) in which phloretin administration was reported to alleviate hepatic steatosis as well as dyslipidemia in mice fed with high-fat diet (Liou et al., 2020). Hepatic lipid buildup causes oxidative stress and the release of proinflammatory cytokines, which culminates in steatohepatitis (Hendrikx and Binder, 2020). Increased concentrations of circulatory pro-inflammatory cytokines viz. IL-6 and TNF-α were documented in the patient with NASH (Bocsan et al., 2017). According to the findings of the current investigation, WD feeding increased the levels of circulating inflammatory cytokines IL-6 and TNF-α as well as their hepatic gene expression, which was effectively reversed by the administration of phloretin (Figures 2E–G), indicating protective response of phloretin against hepatic inflammation. Furthermore, diet-induced gut dysbiosis changed nutritional absorption in the intestine as well as compromised intestinal permeability. This dysregulation increased the supply of nutrients, endotoxins, and microbial metabolites to the liver, resulting in increased hepatic fat deposition as well as increased inflammation (Jiang et al., 2020). It has recently been reported that the intake of WD for an extended period increases the level of endotoxin in the feces of mice (Lee et al., 2020b). Our findings revealed a time-dependent increase in the endotoxin levels in the feces of mice following prolonged exposure to a WD; however, phloretin supplementation could reversed these changes indicating its potential to influence the gut flora.

Recent investigations have established the link between gut dysbiosis and associated changes in microbial signatures and pathogenesis of NAFLD (Safari and Gérard, 2019; Astbury et al., 2020; Lee et al., 2020a; Li et al., 2021). However, very little emphasis was provided to study the time dependent changes in gut microbiota. Next generation sequencing and analysis of comparative microbial diversity and richness revealed significant changes in WD fed mice as compared to control. Despite minor differences in the phloretin treatment group over time, it has maintained the overall diversity index similar to the control group (Figure 3). The findings indicate that the WD has the potential to impact the diversity of gut microbes and might influence the progression of MAFLD by reducing the abundance of advantageous bacteria, while increasing the occurrence of detrimental bacteria linked to a higher risk of MAFLD. The results further surmises the role of phloretin treatment in resisting WD induced changes in microbial diversity and outlined its prophylactic effects.

To delve deeper into dynamics of gut microbiota abundance and distribution, taxonomic signatures were identified among groups. We observed that with increase in time, abundance of *Desulfobacterota* was decreased in the phloretin treatment group similar to control but dissimilar to the WD fed mice (Figure 4A). Prior investigations have revealed an increased abundance of *Desulfobacterota* in NAFLD (Bashiardes et al., 2016; Li et al., 2019) and also implicated in the activation of systemic inflammation (Shi et al., 2021). Increased abundance of *Desulfobacterota* in the WD group can be attributed to high fat diet mediated elevation in endotoxin levels and associated changes in inflammatory markers. Additionally, mean relative abundance of the top 20 families (Figure 4B) revealed an increased in *Muribaculaceae, Tannerellaceae, Desulfovibrionaceae*, and *Lachnospiraceae*, in the WD group similar to the earlier reports (Shen et al., 2017; Li et al., 2019; Hullar et al., 2021). Increased abundance of these obligate anaerobes responsible for production of toxic byproducts and ethanol indicate the ability of WD to induce gut dysbiosis. Moreover, similar to prior finding linking *Peptostreptococcus* to NASH in morbidly obese individuals (Sookoian et al., 2020), our findings revealed higher abundance of *Peptostreptococcus* in MAFLD mice. The family *Ruminococcaceae* and *Lactobacillaceae* was found to be substantially more abundant in the control and phloretin treated groups than in the WD group. The family *Ruminococcaceae* were shown to be more abundant in healthy individuals than in people with NAFLD, and their abundance decreased as the severity of fibrosis worsened (Jiang et al., 2015; Lee et al., 2020a). The importance of phloretin in reversing WD-induced alterations in microbial communities at the phylum and family levels was demonstrated by these findings.

Further, IndVal analysis of taxa (at family, genus and species level) across the treatment groups revealed an increase in the abundance of *Blautia, Terrisporobacter, Alloprevotella, Eubacterium, Clostridium_sensu_stricto, Parabacteroides, Allobaculum*, and *Paludibacetraceae* in WD fed mice indicating a positive association with the progression of MAFLD (Figure 4C). *Blautia* is associated with visceral fat accumulation and found highly abundant in NAFLD and/or NASH conditions including pediatric NAFLD (Safari and Gérard, 2019; Aron-Wisnewsky et al., 2020). Some of these taxa have previously been associated with hepatic damage, characterized by elevated transaminase levels, steatosis, lobular inflammation, and ballooning (Milton-laskibar et al., 2021). Increased abundance of these taxa in WD group is in correlation with the altered pathophysiology, disturbed homeostasis and aberrant lipid metabolism (Figures 1, 2). Additionally, High abundance of anaerobic pathogenic taxa like *Terrisporobacter, Clostridium*, and *Peptostreptococcaceae* in WD groups clearly indicate the ability of WD induced gut dysbiosis to promote the growth of pathogenic microbes in MAFLD condition. Low abundance of these taxa in control and phloretin treated groups indicated that phloretin treatment might have a deleterious impact on their populations. Furthermore, in WD + P group, restored high abundance for *Lactobacillus, Prevotellaceae, Alloprevotella-1, Odoribacter-1, Ruminococcus*, and *Bacteroides-1* was observed, which was lost in WD group. Earlier studies have demonstrated a decrease in the abundance of family *Prevotellaceae* and genus *Lactobacillus*, *Odoribacter* in patients as well as in high fat diet-induced NAFLD (Boursier et al., 2016; Astbury et al., 2020; Pan et al., 2020; Milton-laskibar et al., 2021). Moreover, supplementation with *Lactobacillus* (*Lactobacillus acidophilus, Lactobacillus fermentum*, and *Lactobacillus plantarum*) lowers the progression of non-alcoholic steatosis in mice fed on WD (Lee et al., 2021b). However, genetic heterogeneity between strains at the genomic level can potentially have an impact on the disease state (Yan et al., 2020). According to our findings, different strains of the genus *Odoribacter, Alloprevotella*, and *Lactobacillus* have both favorable and detrimental effects on the pathophysiology of MAFLD (Figure 5). Along with it, *B. thetaiotaomicron*, a common component of gut microflora and an opportunistic pathogen was found in high abundance in WD + P group in fourth week, possibly due to the effect of WD challenge and attain low abundance with the progress of treatment in week 16. Further analysis of community co-occurrence and functional potential associated with these taxa support the microbial abundance observations. *R. flavefaciens* and *Odoribacter* both have been found to be beneficial against NAFLD (Iwao et al., 2020; Pan et al., 2021). These two taxa played a crucial role in maintaining community structure by increasing neighborhood connectivity; their absence in the co-occurrence network plot, on the other hand, resulted in a decrease in neighborhood connection (Figure 6). Previous studies have well reported that *Ruminococcaceae* is less abundant in the patients with progressive NAFLD than in healthy controls and serves as a major component of the microbiota in healthy individuals by preserving the homeostasis of the gut milieu (Qin et al., 2014; Lee et al., 2020a; Tsai et al., 2020). Furthermore, *Ruminococcaceae* family has been shown to effectively alter the microbial composition of NAFLD toward a healthy gut microbiome through microbial interactions (Wu et al., 2020). These findings suggested that they could be used as strong probiotic supplement to help repair the structure of the gut microbial population in patients with NAFLD. Additionally, shift in gut bacterial diversity results in an alteration in the functional profile of bacterial communities as well. The WD group was highly enriched of taxa associated with FA biosynthesis and elongation, which could aggravate the MAFLD condition by promoting both hepatic lipid accumulation and inflammation (Kessler et al., 2014). In addition, long-term WD consumption led to an increase in bile acid biosynthesis pathways, which have been linked to MAFLD in the past (Mouzaki et al., 2016). TCA cycle pathways remained more dominant in WD group. Diet-induced hepatic insulin resistance and fatty liver have been attributed to an elevated TCA cycle (Satapati et al., 2012). Furthermore, WD caused disruption in retinol metabolism pathway which plays a critical role MAFLD progression (Saeed et al., 2021). Along with it, dysregulation of amino acid metabolism pathways has been linked to insulin resistance and altered liver metabolism in patients with liver fibrosis (Hasegawa et al., 2020). Our findings are consistent with previous study indicating that the majority of KEGG pathways associated with NASH and fibrosis patients were involved in the metabolism of carbohydrate, lipid, and amino acids (Boursier et al., 2016). Cytochrome P450 enzymes, found primarily in the liver, are responsible for the metabolism of potentially hazardous substances such as medications, xenobiotics, FAs, and bilirubin (Esteves et al., 2021). Pathways associated with Cytochrome P450 activity were also suppressed by long-term WD feeding. Nevertheless, WD-induced alterations in these metabolic pathways were prevented by phloretin supplementation (Figure 7). Observed high abundance of beneficial microbes and low abundance of pathogenic taxa is a characteristic of healthy gut, which is clearly visible in control and the phloretin treated group. These findings clearly indicate that WD intake alters the gut microbiota, leading to the accumulation of harmful pathogenic microbes with altered metabolic functions, which ultimately resulted in MAFLD progression. Phloretin treatment not only reversed this gut dysbiosis but also favored taxonomic signatures of beneficial microbes thereby slowing course of the disease in the long run. Furthermore, fecal microbiota transplantation (FMT), a method for altering the host microbiome and reversing gut microbiota dysbiosis toward eubiosis, is also widely used these days (Xue et al., 2022). The FMT from mice that had been given phloretin showed a positive effect on pre-clinical ulcerative colitis through modifying the gut microbial community (Wu et al., 2019). These findings strongly suggest that phloretin may have a therapeutic impact on MAFLD through altering the gut microbiota composition.

## 5. Conclusion

**FIGURE 8:**
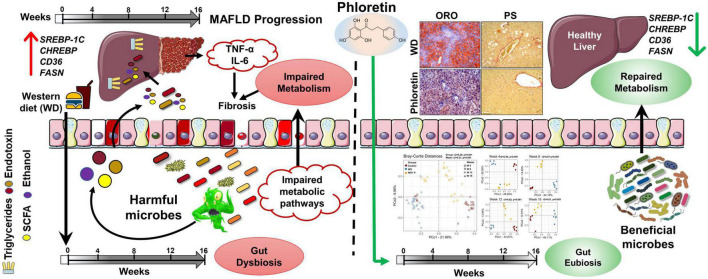
Graphical summary of the work.

In summary (Figure 8), prolonged consumption of WD affects the alpha and beta diversity of gut microbiota as disease progresses. Further, WD feeding resulted in the accumulation of detrimental pathogenic microorganisms which exacerbate the disease pathology by increasing the toxic byproduct, e.g., endotoxin. The impact of genomic variation on pathogenicity, as well as their significance in the course of MAFLD, were also demonstrated by our findings. Phloretin administration, on the other hand, ameliorated WD induced liver steatosis, inflammation, fibrosis, and insulin resistance in mice by reshaping the microbial community composition and suppressing endotoxin release in a time-dependent manner. These findings substantiated the application of phloretin as a dietary intervention approach and as a functional food with favorable prebiotic and eubiotic effects against MAFLD. Additionally, findings of this study open doors for new interventional studies utilizing FMT to probe the uses and effects of phloretin and certain beneficial bacteria as symbiotic, either alone or in combination for the better management of the MAFLD.

## Data availability statement

The datasets presented in this study can be found in online repositories. The names of the repository/repositories and accession number(s) can be found below: https://www.ncbi.nlm.nih.gov/, PRJNA791189.

## Ethics statement

The animal study was approved by the Animal Ethical Committee (IAEC) of CSIR-IHBT, India. The study was conducted in accordance with the local legislation and institutional requirements.

## Author contributions

JC and YP conceptualized the study. JC and PA performed the major bench experiments, collection, analysis, and interpretation of the dataset, and writing of the manuscript. PM processed the NGS raw sequence data and performed *in silico* analysis. MS helped in the library preparation. VP performed the histopathology analysis. RP performed the MiSeq 16S rRNA sequencing and analysis, study-coordination, and writing of the manuscript. RP and YP edited the final version of the manuscript. All authors approved the final version of the manuscript.
