# Supplementary material for: Metagenomic signatures reveal the key role of phloretin in amelioration of gut dysbiosis attributed to metabolic dysfunction-associated fatty liver disease by time-dependent modulation of gut microbiome
Source: Front Microbiol. 2023 Sep 7;14:1210517. doi: 10.3389/fmicb.2023.1210517 (PMC10516607; doi:10.3389/fmicb.2023.1210517)
Supplement: Supplementary file 1 [file Data_Sheet_1.PDF]

Supplementary Information

Metagenomic signatures reveal the key role of Phloretin in amelioration of gut dysbiosis attributed to metabolic-dysfunction associated fatty liver disease (MAFLD) by time-dependent modulation of gut microbiome

Jyoti Chhimwal[1,3], Prince Anand[1,3], Priyanka Mehta[4], Mohit Kumar Swarnkar[2], Vikram Patial[1,3], Rajesh Pandey[3,4*], Yogendra Padwad[1,3*]

[1]*Pharmacology and Toxicology Laboratory, Dietetics and Nutrition Technology Division, CSIR-Institute of Himalayan Bioresource Technology (CSIR-IHBT), Palampur-176061, India*

[2]*Biotechnology Division, CSIR-Institute of Himalayan Bioresource Technology (CSIR-IHBT), Palampur-176061, India*

[3]*Academy of Scientific and Innovative Research (AcSIR), Ghaziabad-201002, India.*

[4]*INtegrative GENomics of HOst-PathogEn (INGEN-HOPE) laboratory, CSIR-Institute of Genomics and Integrative Biology (CSIR-IGIB), Mall Road, Delhi 110007, India*

[*]Corresponding authors:

Yogendra Padwad (yogendra@ihbt.res.in, Tel (off): +91-1894-233339; Extn. 473; (self): +919816747680); ORCID: https://orcid.org/0000-0003-1793-9340

Rajesh Pandey (rajeshp@igib.in, Tel (self): 9811029551; ORCID: https://orcid.org/0000-0002-4404-8327

Authors' information

Jyoti Chhimwal: jyoti24mar@gmail.com; ORCID: https://orcid.org/0000-0002-6155-7721

Prince Anand: princeanandsaharsa4u@gmail.com; ORCID: https://orcid.org/0000-0003-3004-5923

Priyanka Mehta: priyanka.m@igib.in; ORCID: https://orcid.org/0000-0001-6298-4322

Mohit Kumar Swarnkar: mohitks@ihbt.res.in; ORCID: https://orcid.org/0000-0002-7033-2364

Vikram Patial: vikrampatial@ihbt.res.in; ORCID: https://orcid.org/0000-0002-4912-9871

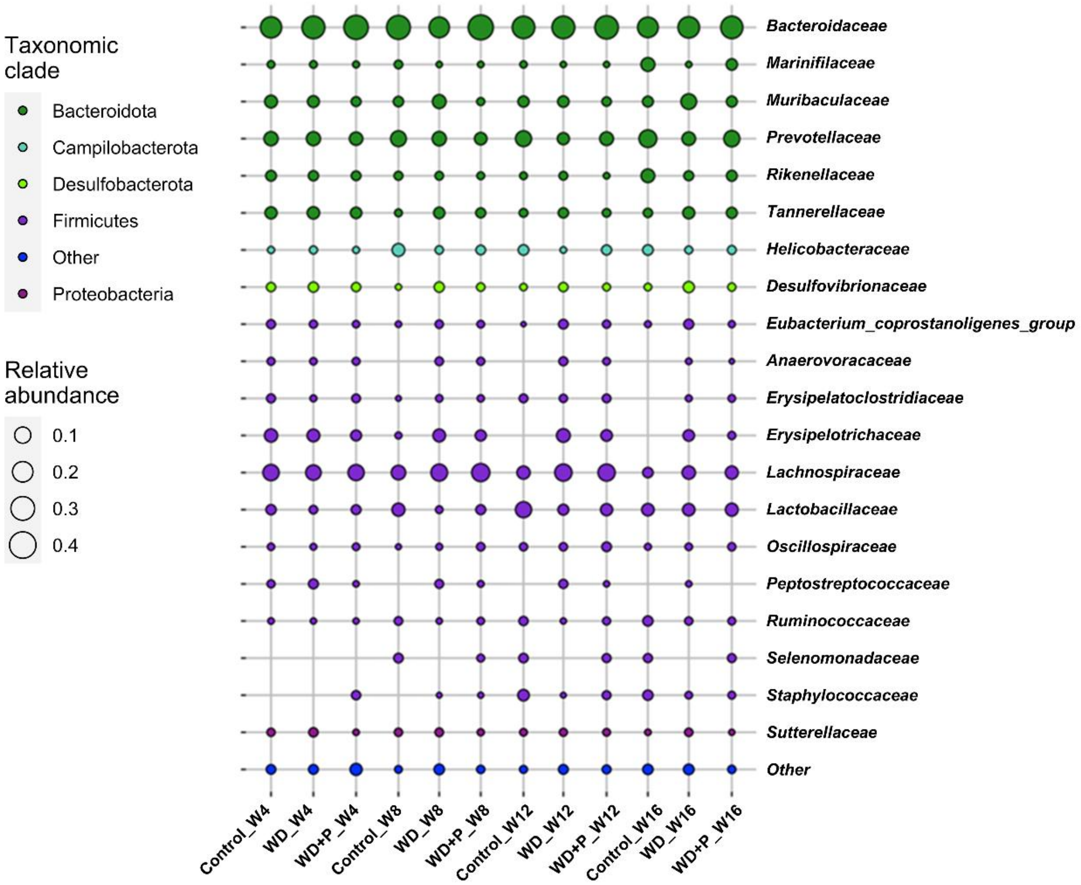

Figure S1: Bubble plot representing the bacterial community composition at family level grouped by week

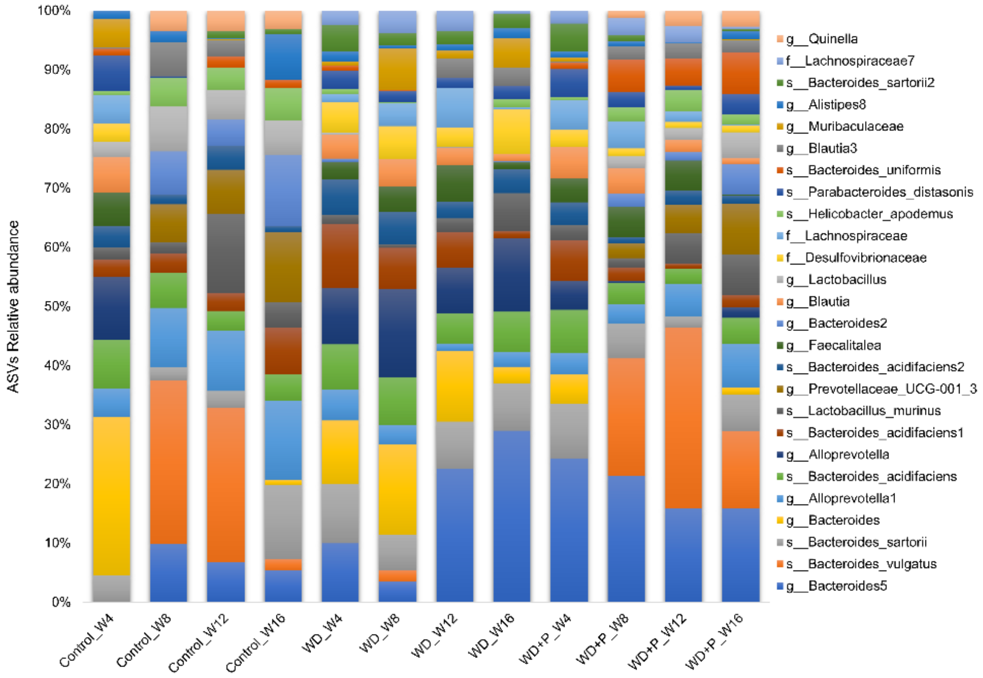

Figure S2: Bar chart representing the bacterial community composition at ASV level

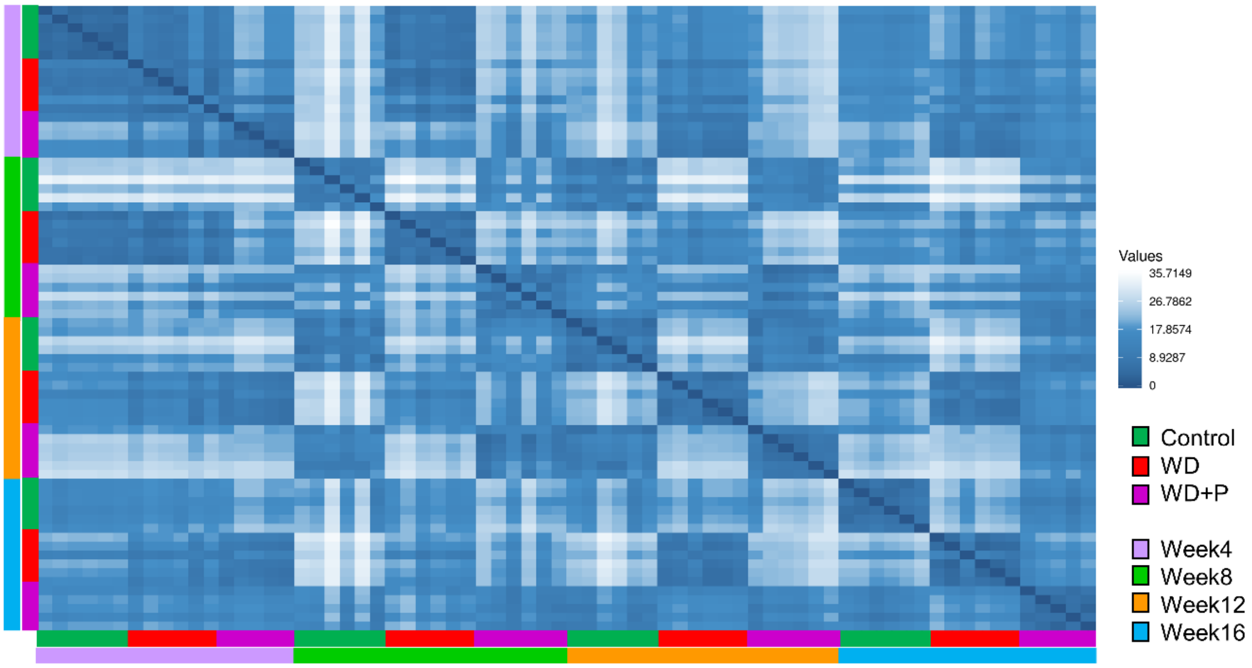

Figure S3: Heatmap signature of Spearman rank correlation between samples calculated using Euclidean distance

Table S1: List of primer sequences used for qRT-PCR

Gene	Mouse rimer sequences (5′ end to 3′ end)	
	**Forward**	**Reverse**
CD36	CTCCTGTTCACTGTGGGTCC	AGAGCATTGTGCCAGAGGTC
ChREBP	GATGGTGCGAACAGCTCTTCT	CTGGGCTGTGTCATGGTGAA
IL-6	CTTCCATCCAGTTGCCTTCTTG	GGGAGTGGTATCCTCTGTGAAGTC
FASN	CACCCACTGGAGCTGGTAT	TCGAGGAAGGCACTACACCT
PPAR-α	CGGGAAAGACCAGCAACAAC	TGGCAGCAGTGGAAGAATCG
PPAR-γ	TCGCTGATGCACTGCCTATG	TGTCAAAGGAATGCGAGTGGTC
SREBP-1c	GATGTGCGAACTGGACACAG	CATAGGGGGCGTCAAACAG
TNF-α	GAAACACAAGATGCTGGGACAGT	CATTCGAGGCTCCAGTGAATTC
GAPDH	TCACTCAAGATTGTCAGCAATGC	TCATGAGCCCTTCCACAATG

Table S2: Shannon and Simpson indices of individual samples in each group with sample ID

Sample ID	Group	No. of reads	Shannon	InvSimpson	Observed ASV	Obs ASV.rarified
X1	Control_W4	24,679	3.320511	14.54429	49	48.99426
X2	Control_W4	38,278	3.704854	22.5794	74	72.93069
X3	Control_W4	41,252	3.679278	23.71001	69	68.0399
X4	Control_W4	25,047	3.311709	15.31917	49	48.56997
X5	Control_W4	30,857	3.598345	24.90901	56	55.90923
X6	Control_W4	22,010	3.328972	15.97795	48	47.97018
X7	WD_W4	33,246	3.670094	27.56711	63	62.41357
X8	WD_W4	35,567	3.618913	27.16032	56	55.30031
X9	WD_W4	27,155	3.776268	32.22004	65	64.10761
X10	WD_W4	32,297	3.807654	32.93378	67	66.54876
X11	WD_W4	32,295	3.533172	22.32914	56	55.72538
X12	WD_W4	24,703	3.520839	24.632	49	48.97119
X13	WD + P_W4	30,179	3.544563	23.2446	59	58.62677
X14	WD + P_W4	28,457	3.300714	14.06442	60	58.99556
X15	WD + P_W4	27,434	3.123869	14.06692	38	37.94018
X17	WD + P_W4	29,272	3.533271	21.16662	57	56.18834
X18	WD + P_W4	25,950	3.462603	19.95079	53	52.75474
X19	Control_W8	41,695	3.293658	15.21652	49	48.9024
X20	Control_W8	29,237	3.049966	14.00763	35	34.99973
X21	Control_W8	31,673	2.661563	8.620677	26	25.99984
X22	Control_W8	20,969	3.182203	16.25119	39	38.99904
X23	Control_W8	33,708	2.757448	9.455838	29	28.99934
X24	Control_W8	31,045	3.333206	20.43491	44	43.9806
X25	WD_W8	30,172	3.677348	24.72984	67	65.49127
X26	WD_W8	30,335	3.662072	25.22194	63	62.24449
X27	WD_W8	34,120	3.782158	32.30495	69	67.23388
X28	WD_W8	29,952	3.527399	22.57344	57	56.69387
X29	WD_W8	33,329	3.705538	26.90463	70	67.91991
X30	WD_W8	34,800	3.783695	31.47046	70	68.35792
X31	WD + P_W8	26,312	3.406886	14.17153	61	60.27967

X32	WD + P_W8	26,372	3.242139	14.58995	49	48.47057
X33	WD + P_W8	34,125	3.615576	21.66507	65	63.62467
X34	WD + P_W8	28,136	3.243898	12.47218	57	55.49794
X35	WD + P_W8	40,570	3.498437	18.67041	62	61.42243
X36	WD + P_W8	36,519	3.214475	16.54278	41	40.58032
X37	Control_W12	37,264	3.410143	18.44227	53	52.89001
X38	Control_W12	32,551	3.400953	21.18677	46	45.01857
X39	Control_W12	34,200	3.167283	12.20761	48	47.78306
X40	Control_W12	26,840	3.220278	14.17384	45	44.57278
X41	Control_W12	36,280	3.35492	19.05334	49	48.9704
X42	Control_W12	28,390	3.208388	14.22206	46	45.51885
X43	WD_W12	34,967	3.396866	19.56449	47	46.74587
X44	WD_W12	29,604	3.031236	14.94175	32	32
X45	WD_W12	42,786	3.446522	21.67568	50	49.28993
X46	WD_W12	36,290	3.537983	21.30396	58	57.51846
X47	WD_W12	37,976	3.55584	21.59899	58	57.65603
X48	WD_W12	35,001	3.361079	19.86302	42	41.89357
X49	WD + P_W12	37,505	3.676856	24.62932	68	65.90462
X50	WD + P_W12	32,504	3.355392	14.08054	61	60.57658
X51	WD + P_W12	39,408	3.422862	15.56476	62	60.83228
X52	WD + P_W12	37,496	3.325866	15.07722	52	51.72279
X53	WD + P_W12	39,177	3.366982	13.2069	63	61.48576
X54	WD + P_W12	36,198	3.328035	11.79292	66	63.82564
X55	Control_W16	38,469	3.577482	25.16181	57	56.91669
X56	Control_W16	29,663	3.384728	21.66135	44	43.87965
X57	Control_W16	38,354	3.469381	24.68763	47	46.74309
X58	Control_W16	38,657	3.42885	24.25922	46	45.10131
X59	Control_W16	38,154	3.350001	21.98234	42	41.74934
X60	Control_W16	41,307	3.152228	18.88926	32	31.99969
X61	WD_W16	35,673	3.175202	14.04022	42	41.8329
X62	WD_W16	36,487	3.453773	18.09187	53	52.65239
X63	WD_W16	33,459	3.512643	27.01736	47	46.15254
X64	WD_W16	39,441	3.301628	15.76386	47	46.51733

X65	WD_W16	52,389	3.713861	20.49163	78	76.72611
X66	WD_W16	45,640	3.654487	22.52544	68	66.5875
X67	WD + P_W16	42,237	3.768838	28.93256	69	67.74327
X68	WD + P_W16	42,419	3.502307	22.59285	59	58.16792
X70	WD + P_W16	89,881	3.738563	25.3435	84	80.73335
X71	WD + P_W16	43,464	3.881051	33.54251	76	74.78241
X72	WD + P_W16	32,165	3.338684	20.20713	47	46.35472

Table S3: Variance partitioning of microbial taxa with respect to diet pattern and weeks

ASV	Diet Pattern	Week	Residuals
g__Parabacteroides	0.75091911	0	0.24908089
g__Muribaculaceae23	0.61740716	0.08673617	0.29585667
f__Paludibacteraceae	0.58000796	0.01729516	0.40269689
g__Clostridium_sensu_stricto_1	0.57768033	0.04125556	0.38106411
s__Bacteroides_nordii	0.56528364	0.01041569	0.42430067
f__Desulfovibrionaceae	0.52443844	0.11067664	0.36488492
g__Alloprevotella1	0.50119449	0.04583826	0.45296725
s__Bacteroides_uniformis	0.49700741	0.02002182	0.48297076
g__Lactobacillus	0.48787663	0.10079302	0.41133035
g__Alloprevotella	0.46938853	0.14140827	0.38920321
g__Prevotellaceae_Ga6A1_1	0.46034899	1.18E-09	0.53965101
f__Lachnospiraceae7	0.45816141	0.22812748	0.31371111
g__Blautia4	0.39669537	0.09547547	0.50782916
g__Prevotellaceae_UCG.001_3	0.39425148	0.29756144	0.30818708
g__Bacteroides2	0.36962099	0.25087618	0.37950283
g__Bacteroides5	0.36920885	0.00903941	0.62175174
g__Eubacterium_coprostanoligenes	0.3596458	0.02717481	0.61317939
g__Eubacterium_coprostanoligenes1	0.35873425	0.03233077	0.60893498
g__Terrisporobacter	0.35008727	0.14868088	0.50123186
g__Blautia1	0.34646309	0.14575471	0.5077822
g__Odoribacter	0.31283549	0.13796305	0.54920146
g__Quinella	0.29898064	0.17295212	0.52806724

g__Ruminococcus_torques2	0.2974495	0.07921947	0.62333103
s__Bacteroides_vulgatus	0.29502098	0.3665007	0.33847832
g__Hungatella	0.27886333	0.21293615	0.50820052
g__Allobaculum2	0.27483556	0.09175818	0.63340626
g__Odoribacter1	0.27058877	0.26331748	0.46609375
s__Bacteroides_nordii1	0.26965954	0	0.73034046
s__Bacteroides_thetaiotaomicron	0.26928192	0.0998975	0.63082058
s__Lactobacillus_johnsonii	0.26384714	0.06426179	0.67189107
g__Allobaculum3	0.2611881	0.1266565	0.61215541
g__Muribaculaceae7	0.25810526	0.06698178	0.67491296
g__Lachnoclostridium	0.25451755	0.1930374	0.55244505
s__Bacteroides_caecimuris1	0.25290232	0.02880526	0.71829242
g__Eubacterium_nodatum	0.25030847	0.21385498	0.53583655
s__Helicobacter_apodemus	0.24269678	0.08729969	0.67000353
g__Prevotellaceae_UCG.001_1	0.236604	0.11728204	0.64611396
g__Romboutsia	0.23572831	0.03065647	0.73361522
g__Muribaculaceae	0.22914843	0.04860657	0.722245
s__Bacteroides_acidifaciens2	0.22698183	0.22881918	0.54419899
g__.Ruminococcus._torques	0.20892182	1.15E-10	0.79107818
g__Prevotellaceae_Ga6A1_2	0.19998188	0.00283478	0.79718334
g__Faecalibaculum	0.19973008	0.21901147	0.58125845
s__Ruminococcus_flavefaciens	0.19552033	0.20360026	0.60087941
g__Muribaculaceae2	0.19393688	0.0056987	0.80036442
s__Clostridium_paraputrificum	0.19263265	0.10707025	0.7002971
g__Muribaculaceae18	0.19235751	0.12371702	0.68392548
g__Prevotellaceae_UCG.001_4	0.19076462	0.27909201	0.53014337
f__Lachnospiraceae9	0.18913349	0.16400939	0.64685712
s__Bacteroides_sartorii2	0.18862476	0.15163117	0.65974407
g__Muribaculaceae14	0.18562082	0.02398918	0.79039
s__Anaerostipes_caccae	0.18478474	0.24160303	0.57361223
g__Muribaculaceae9	0.18281674	0	0.81718326
s__Burkholderiales_bacterium1	0.18215944	0.04239067	0.77544989
f__Lachnospiraceae1	0.18137424	0.09614163	0.72248412

g__Muribaculum	0.17755993	0.19961928	0.62282079
s__Bacteroides_uniformis2	0.1689706	0.1996153	0.6314141
g__Muribaculaceae25	0.16476048	0.15848014	0.67675938
s__Dubosiella_newyorkensis	0.16225386	0.3100645	0.52768164
g__Alistipes1	0.16191762	0.21340683	0.62467555
s__Holdemania_massiliensis	0.16184514	0.01185713	0.82629773
f__Oscillospiraceae	0.1594598	0.0786095	0.7619307
g__Clostridia_UCG.014	0.15843921	0.14992818	0.69163261
g__Faecalitalea	0.15822037	0.27470347	0.56707616
g__Bacteroides	0.15249182	0.31390211	0.53360607
f__Oscillospiraceae1	0.14977686	0.32853139	0.52169175
s__uncultured_Bacteroidales	0.14202194	0.01635189	0.84162617
g__Alistipes8	0.14098374	0.2553182	0.60369806
g__Blautia	0.13582221	0.47211049	0.3920673
g__Ruminococcus_torques1	0.13530062	0.01623722	0.84846216
g__Lachnospiraceae_NK4A136_group3	0.13470359	0.0901358	0.77516061
g__Staphylococcus	0.13250568	0.18241239	0.68508193
g__Bacteroides4	0.12911033	0.09960797	0.77128169
g__Lachnoclostridium1	0.12663477	0.25652793	0.6168373
s__Bacteroides_acidifaciens	0.12660962	0.47134618	0.4020442
g__Ruminococcus_gnavus	0.12557144	0.24437767	0.63005089
g__Muribaculaceae19	0.12451912	0	0.87548088
g__Tyzzerella	0.12298353	0.16601079	0.71100568
g__Oscillibacter	0.11752993	0.11613535	0.76633472
s__Erysipelatoclostridium_ramosum1	0.11723127	0.03875382	0.84401491
f__Lachnospiraceae6	0.11665379	0.23093499	0.65241121
g__Odoribacter4	0.11574743	0.41043056	0.47382201
g__Prevotellaceae_UCG.001_2	0.11377635	0.24083605	0.6453876
g__Allobaculum6	0.11265344	0.08184816	0.8054984
s__Burkholderiales_bacterium	0.11026753	0.18792284	0.70180962
g__Butyricimonas	0.10987631	0.22652467	0.66359902
g__ASF356_2	0.10850226	0.07998213	0.81151561
f__Desulfovibrionaceae1	0.10839899	0.07571052	0.81589049

s__Acinetobacter_variabilis	0.10761248	0.37434838	0.51803914
o__Rhodospirillales1	0.10738936	0.11567319	0.77693745
g__Erysipelatoclostridium1	0.10658357	0.02947342	0.86394301
s__Bacteroides_acidifaciens1	0.10357386	0.12637013	0.77005601
g__Faecalitalea1	0.10018532	0.01736587	0.88244882
g__Negativibacillus	0.09966329	1.58E-10	0.90033671
g__Eubacterium_coprostanoligenes2	0.09794469	0.06647613	0.83557918
g__Allobaculum7	0.09668771	0.00095075	0.90236154
g__Paraprevotella	0.09659721	0.07235442	0.83104837
g__Escherichia.Shigella	0.09543888	0.24973187	0.65482925
g__Bacteroides3	0.09318614	0	0.90681386
g__Ruminococcus_gauvreauii_group	0.09213863	0.07274027	0.8351211
g__Muribaculaceae21	0.09192923	0.08787574	0.82019503
g__Rikenella	0.08995467	0.09604269	0.81400264
g__Eubacterium_fissicatena	0.08863506	0.22841834	0.6829466
g__Blautia2	0.08830657	0.14652459	0.76516884
g__Muribaculaceae15	0.08739475	0.15246139	0.76014386
s__Parabacteroides_distasonis4	0.08733887	0.07735929	0.83530184
g__Alistipes5	0.08680663	0.03936111	0.87383226
g__Clostridium_innocuum_group	0.0867896	0.12776409	0.78544631
s__Staphylococcus_lentus	0.08603627	0.08405089	0.82991284
g__Lachnospiraceae_NK4A136_group2	0.08465651	0.15255891	0.76278458
g__Butyricicoccus1	0.07996891	0.11999823	0.80003286
s__Bacteroides_uniformis1	0.07892753	0.05710627	0.8639662
s__Helicobacter_typhlonius	0.07865946	0.09707063	0.82426991
g__Ruminococcus	0.07796828	0.21537951	0.70665221
g__Lactobacillus1	0.07720437	0.17520744	0.74758818
g__Prevotellaceae_NK3B31_group	0.07695118	0.10504461	0.81800421
f__Lachnospiraceae2	0.07689883	0.19796312	0.72513805
g__Anaerotruncus1	0.07673983	0.15595462	0.76730555
s__Lactobacillus_murinus	0.07511628	0.27853795	0.64634577
f__Lachnospiraceae	0.07474346	0.16871412	0.75654242
o__Rhodospirillales	0.07135468	0.16297225	0.76567307

g__Faecalitalea2	0.07104726	0.17768848	0.75126426
g__Paraprevotella3	0.07016993	0.05813939	0.87169069
s__Streptococcus_hyointestinalis	0.06992033	0.10940953	0.82067015
g__Alistipes6	0.0685005	0.08000685	0.85149265
g__Flavonifractor	0.06709176	0.41194517	0.52096307
g__Holdemania	0.06484796	0	0.93515204
g__Eubacterium_siraeum	0.06113955	0.29647156	0.64238889
g__Allobaculum	0.06089409	0.11043259	0.82867332
s__Parabacteroides_distasonis1	0.0598412	0	0.9401588
s__Burkholderiales_bacterium2	0.05978631	0.09081053	0.84940316
s__Bacteroides_sartorii	0.05865742	0.22827466	0.71306792
g__Odoribacter2	0.05844267	0.33267917	0.60887816
g__Gastranaerophilales	0.05708176	0.09480311	0.84811513
g__Anaerotruncus	0.05452766	0.02113893	0.92433341
g__Muribaculaceae10	0.05445968	0.28872092	0.6568194
g__Parabacteroides3	0.05350837	0.15641151	0.79008012
g__Muribaculaceae13	0.05333536	0.1792583	0.76740633
g__Lachnospiraceae_NK4A136_group1	0.0512153	0.11431624	0.83446845
g__Roseburia	0.05005259	0.05436978	0.89557764
g__Paraprevotella4	0.04911102	0.01668812	0.93420086
s__Parabacteroides_distasonis	0.04684596	0.32819353	0.62496051
g__Parabacteroides1	0.04602017	3.69E-10	0.95397983
f__Lachnospiraceae3	0.04560104	0.04110623	0.91329273
g__Jeotgalicoccus	0.04484735	0.01339225	0.9417604
f__Erysipelatoclostridiaceae	0.04430511	0.01352928	0.94216561
g__Muribaculaceae24	0.04422701	0.16245129	0.7933217
s__Lactobacillus_reuteri	0.04090161	0.06317022	0.89592817
s__Coprobacillus_cateniformis	0.04023739	0.2113954	0.74836721
g__Aerococcus	0.04021557	0.10336716	0.85641728
g__Erysipelatoclostridium	0.04005406	0.08729593	0.87265001
f__Lachnospiraceae4	0.0395973	0.02033647	0.94006623
g__Allobaculum5	0.03717407	0.04839876	0.91442717
g__Muribaculaceae4	0.03583638	0.06934661	0.89481702

g__Muribaculaceae6	0.03557046	0.09948539	0.86494415
f__Lachnospiraceae8	0.03429385	0.02624634	0.93945981
g__Alistipes2	0.0336267	0.00929584	0.95707746
g__Allobaculum1	0.03264463	0.06253062	0.90482475
s__Parabacteroides_distasonis3	0.03187584	0.0611547	0.90696946
s__Erysipelatoclostridium_ramosum	0.03127378	0.0138875	0.95483872
s__Parabacteroides_distasonis5	0.03102527	0.16469715	0.80427757
g__Kurthia	0.03044445	0.1149662	0.85458935
g__Rikenellaceae_RC9_gut_group2	0.02819067	0.1657762	0.80603313
g__Colidextribacter	0.02704546	0.07992438	0.89303016
g__Butyricimonas2	0.02604954	0	0.97395046
s__Lactobacillus_reuteri1	0.02567353	0.15455443	0.81977203
g__Alistipes7	0.02554955	0.0632538	0.91119664
g__Bacteroides1	0.02468949	0.06730057	0.90800995
g__ASF356_1	0.02468945	0.01142266	0.96388789
g__Muribaculaceae20	0.02447494	0.07059686	0.9049282
g__Alistipes9	0.0244272	0.02833582	0.94723698
g__Muribaculaceae8	0.02364039	0.06600515	0.91035446
f__Lachnospiraceae10	0.02276162	0.15243781	0.82480057
g__Rikenellaceae_RC9_gut_group3	0.02275383	0.16266225	0.81458392
g__UBA1819	0.02218147	0.0716843	0.90613423
g__Rikenellaceae_RC9_gut_group1	0.02195576	0.00969368	0.96835056
g__Muribaculaceae3	0.01925838	0.07871955	0.90202207
g__Alistipes4	0.0155734	0	0.9844266
g__Muribaculaceae11	0.01480893	0.2378707	0.74732037
g__Alistipes3	0.01380971	0.07717161	0.90901868
g__Muribaculaceae17	0.01264917	0.05557921	0.93177162
f__Lachnospiraceae5	0.01137266	0.05143356	0.93719377
s__Lactobacillus_murinus1	0.01086214	0.08493487	0.90420299
g__Paraprevotella1	0.01069188	0	0.98930812
g__Atopostipes	0.01058945	0.07791872	0.91149183
s__Bacteroides_sartorii1	0.00903489	0.06206663	0.92889849
s__Burkholderiales_bacterium3	0.00628914	0	0.99371086

g__Blautia3	0.00347715	0.23008103	0.76644182
g__Helicobacter	0.00342222	0.28459245	0.71198533
g__Candidatus_Stoquefichus1	0.00247038	0.04487696	0.95265266
g__Muribaculaceae1	0.0019312	0.09195271	0.90611609
g__Candidatus_Stoquefichus	3.64E-10	0.04531773	0.95468227
g__Muribaculaceae22	8.91E-16	0	1
g__Muribaculaceae12	8.13E-18	0.11877477	0.88122523

Table S4: ASV Indval summary and P value of taxa from each group

**Taxa**	**Group**	**IndVal**	**P value**	**freq.**
g__Lactobacillus	Control	0.70345592	0.001	43
g__Alloprevotella1	Control	0.55155547	0.001	69
g__Bacteroides2	Control	0.53002048	0.001	39
g__Odoribacter1	Control	0.51234322	0.001	32
s__Bacteroides_caecimuris1	Control	0.46504594	0.001	31
g__Prevotellaceae_UCG.001_3	Control	0.45881929	0.001	34
s__Helicobacter_apodemus	Control	0.43124962	0.004	41
g__Alistipes8	Control	0.42671737	0.025	52
g__Quinella	Control	0.39979553	0.005	31
g__Prevotellaceae_UCG.001_4	Control	0.3837467	0.001	13
s__Ruminococcus_flavefaciens	Control	0.381106	0.001	19
g__Prevotellaceae_UCG.001_1	Control	0.34509138	0.008	23
g__Alistipes1	Control	0.32082802	0.011	21
g__Muribaculaceae18	Control	0.31143634	0.007	18
s__Anaerostipes_caccae	Control	0.30621571	0.002	16
s__Bacteroides_uniformis2	Control	0.28449664	0.016	16
g__ASF356_2	Control	0.270955	0.011	13
g__Lachnospiraceae_NK4A136_group3	Control	0.26597159	0.001	8
s__Erysipelatoclostridium_ramosum	Control	0.25248564	0.041	19
f__Lachnospiraceae3	Control	0.24945917	0.02	13
g__Oscillibacter	Control	0.23923511	0.016	12
g__Ruminococcus	Control	0.23826535	0.028	14

g__Eubacterium_coprostanoligenes2	Control	0.2349691	0.003	8
g__Prevotellaceae_NK3B31_group	Control	0.23235452	0.046	16
g__Odoribacter4	Control	0.22675149	0.03	13
g__Butyricicoccus1	Control	0.21665588	0.022	10
f__Lachnospiraceae6	Control	0.20555306	0.015	8
g__Alistipes5	Control	0.19773945	0.011	6
g__Butyricimonas	Control	0.1916609	0.011	6
s__Burkholderiales_bacterium	Control	0.18760382	0.015	6
s__Helicobacter_typhlonius	Control	0.18628773	0.046	10
s__Acinetobacter_variabilis	Control	0.18572134	0.035	9
f__Lachnospiraceae2	Control	0.18056998	0.044	9
g__Escherichia.Shigella	Control	0.17638842	0.017	6
g__Parabacteroides	WD	0.92111799	0.001	27
g__Clostridium_sensu_stricto_1	WD	0.9048986	0.001	28
f__Paludibacteraceae	WD	0.83804289	0.001	25
g__Muribaculaceae23	WD	0.75568973	0.001	41
g__Alloprevotella	WD	0.70387706	0.001	42
g__Eubacterium_coprostanoligenes	WD	0.67218958	0.001	33
s__Lactobacillus_johnsonii	WD	0.63747577	0.001	22
f__Desulfovibrionaceae	WD	0.61438961	0.001	42
g__Muribaculaceae	WD	0.57725356	0.001	30
g__Odoribacter	WD	0.55101254	0.001	18
g__Blautia4	WD	0.54992456	0.001	29
g__Bacteroides	WD	0.53582622	0.001	39
g__Blautia1	WD	0.53433785	0.001	17
g__Terrisporobacter	WD	0.50755978	0.001	20
g__Muribaculaceae7	WD	0.49033668	0.001	29
s__Dubosiella_newyorkensis	WD	0.46970989	0.001	20
s__Bacteroides_acidifaciens1	WD	0.45313156	0.007	66
g__Allobaculum2	WD	0.45076238	0.001	12
s__Bacteroides_acidifaciens2	WD	0.45030291	0.001	58
g__Eubacterium_nodatum	WD	0.43932452	0.003	42
s__Bacteroides_sartorii2	WD	0.41989177	0.003	31

g__Allobaculum3	WD	0.41666667	0.001	10
g__Romboutsia	WD	0.41396444	0.002	16
g__Muribaculaceae9	WD	0.4063051	0.001	21
g__Ruminococcus_torques	WD	0.40288263	0.001	16
s__Bacteroides_sartorii	WD	0.39951974	0.031	65
g__Lachnoclostridium	WD	0.39171944	0.003	31
s__Bacteroides_acidifaciens	WD	0.38622979	0.021	70
g__Muribaculaceae2	WD	0.37684744	0.001	20
f__Lachnospiraceae9	WD	0.373755	0.003	21
s__Burkholderiales_bacterium1	WD	0.35378405	0.001	12
g__Bacteroides4	WD	0.34915531	0.009	22
g__Paraprevotella	WD	0.34100623	0.027	34
g__Muribaculum	WD	0.33997004	0.002	16
g__Eubacterium_fissicatena	WD	0.33215649	0.008	26
s__Clostridium_paraputrificum	WD	0.326974	0.001	11
g__Negativibacillus	WD	0.31377782	0.001	13
s__uncultured_Bacteroidales	WD	0.3020014	0.003	10
g__Allobaculum6	WD	0.29166667	0.001	7
g__Muribaculaceae19	WD	0.26824182	0.002	11
g__Paraprevotella3	WD	0.26131359	0.048	22
g__Clostridium_innocuum_group	WD	0.25432071	0.038	19
g__Allobaculum5	WD	0.25	0.002	6
g__Allobaculum7	WD	0.25	0.006	6
o__Rhodospirillales1	WD	0.25	0.008	6
s__Parabacteroides_distasonis4	WD	0.21645154	0.015	7
s__Parabacteroides_distasonis1	WD	0.20212797	0.048	12
f__Erysipelatoclostridiaceae	WD	0.18265969	0.024	6
g__Holdemania	WD	0.1780165	0.015	6
g__Rikenellaceae_RC9_gut_group1	WD	0.14491734	0.048	6
s__Bacteroides_nordii	WD + P	0.69910468	0.001	17
s__Bacteroides_uniformis	WD + P	0.68262905	0.001	30
g__Prevotellaceae_Ga6A1_1	WD + P	0.62325193	0.001	35
g__Eubacterium_coprostanoligenes1	WD + P	0.56361299	0.001	25

g__Hungatella	WD + P	0.5	0.001	11
g__Bacteroides5	WD + P	0.49247507	0.001	62
s__Bacteroides_thetaiotaomicron	WD + P	0.47829688	0.001	13
g__Ruminococcus_torques2	WD + P	0.47608742	0.001	15
g__Ruminococcus_torques1	WD + P	0.42562183	0.002	30
f__Lachnospiraceae7	WD + P	0.41884908	0.001	38
s__Bacteroides_vulgatus	WD + P	0.41143108	0.003	36
s__Bacteroides_nordii1	WD + P	0.40909091	0.001	9
g__Blautia	WD + P	0.39013965	0.014	47
g__Prevotellaceae_Ga6A1_2	WD + P	0.38720645	0.001	13
g__Bacteroides3	WD + P	0.36998506	0.002	15
g__Faecalitalea	WD + P	0.36784774	0.028	45
s__Parabacteroides_distasonis	WD + P	0.32292802	0.038	35
g__Faecalibaculum	WD + P	0.31818182	0.001	7
g__Muribaculaceae14	WD + P	0.31252948	0.001	10
g__Tyzzerella	WD + P	0.3039815	0.005	19
s__Erysipelatoclostridium_ramosum1	WD + P	0.2880133	0.009	13
g__Erysipelatoclostridium1	WD + P	0.284251	0.008	11
f__Lachnospiraceae1	WD + P	0.27272727	0.002	6
f__Oscillospiraceae	WD + P	0.27272727	0.002	6
g__Clostridia_UCG.014	WD + P	0.27272727	0.002	6
g__Faecalitalea1	WD + P	0.27272727	0.002	6
s__Holdemania_massiliensis	WD + P	0.27272727	0.002	6
f__Oscillospiraceae1	WD + P	0.27200249	0.014	17
s__Bacteroides_uniformis1	WD + P	0.21590688	0.009	7
g__Lachnospiraceae_NK4A136_group2	WD + P	0.19846538	0.009	6
